# Alternative promoters in CpG depleted regions are prevalently associated with epigenetic misregulation of liver cancer transcriptomes

**DOI:** 10.1038/s41467-023-38272-4

**Published:** 2023-05-11

**Authors:** Chirag Nepal, Jesper B. Andersen

**Affiliations:** 1 Biotech Research and Innovation Centre (BRIC), Department of Health and Medical Sciences, University of Copenhagen, Ole Maaløes Vej 5, Copenhagen N, DK-2200 Denmark; 2 Center for Genomics, School of Medicine, Loma Linda University, Loma Linda, CA 92350 USA

**Keywords:** Genome informatics, Liver, Liver cancer

## Abstract

Transcriptional regulation is commonly governed by alternative promoters. However, the regulatory architecture in alternative and reference promoters, and how they differ, remains elusive. In 100 CAGE-seq libraries from hepatocellular carcinoma patients, here we annotate 4083 alternative promoters in 2926 multi-promoter genes, which are largely undetected in normal livers. These genes are enriched in oncogenic processes and predominantly show association with overall survival. Alternative promoters are narrow nucleosome depleted regions, CpG island depleted, and enriched for tissue-specific transcription factors. Globally tumors lose DNA methylation. We show hierarchical retention of intragenic DNA methylation with CG-poor regions rapidly losing methylation, while CG-rich regions retain it, a process mediated by differential *SETD2*, H3K36me3, *DNMT3B*, and *TET1* binding. This mechanism is validated in *SETD2* knockdown cells and *SETD2*-mutated patients. Selective DNA methylation loss in CG-poor regions makes the chromatin accessible for alternative transcription. We show alternative promoters can control tumor transcriptomes and their regulatory architecture.

## Abstract (web summary)

The regulatory mechanisms of alternative promoters remain to be investigated. Here, the authors explore the sequence and epigenetics landscape of alternative promoters and how they regulate gene expression in hepatocellular carcinoma.

## Introduction

Hepatocellular carcinoma (HCC) accounts for about 90% of primary liver cancers and is the third leading cause of cancer-related death^1^. Genome-wide profiling of HCC patients has helped to build a molecular map of mutations, dysregulated genes, and DNA methylomes^2–5^. Additional efforts to map histone modifications^6^, chromatin accessibility^7^, transposon activation^8^, and RNA N6-methyladenosine (m6A) methylation^9^ are ongoing and important steps to understanding mechanisms of gene regulation in cancer. Promoters are gateways to start transcription and regulate gene expression in a temporal and spatial manner, but little is known about promoter regulation in cancer. Therefore, we aimed to understand how the regulatory architecture of promoter usage impacts gene regulation using HCC as a model.

Transcription is facilitated by a reference promoter, which is the region proximal to the transcription start site (TSS), integrating *cis*-regulatory elements to ensure precise gene regulation^10^. Cap Analysis of Gene Expression Sequencing (CAGE-seq) determines the 5’-ends of TSSs at single nucleotide resolution^11,12^ and quantifies gene expression similar to RNA-seq^13^. CAGE-seq also detects differently regulated transcription initiation events within the same core promoter^14,15^, and thus, it can accurately detect alternative promoters. In addition to reference promoters, many genes utilize alternative promoters for specific processes, such as cell fate transitions in yeast^16^ and mammalian cells^17^, in vertebrate embryogenesis^12,18^, and may have important roles in cancer^19^. Widespread activation of alternative promoters is known in different contexts; however, it is unclear whether their (epi-)genetic states are different compared to the reference promoter and if alternative promoters are under a different regulatory architecture. Alternative promoters have dynamic intragenic DNA methylation across human tissues^20^, and loss of DNA methyltransferase 3B (*DNMT3B*) in mouse embryonic stem (ES) cells was shown to result in spurious initiation of alternative promoters^21^. Therefore, precise regulation of alternative promoters is important to ensure correct gene expression. Transcription of alternative promoters is widespread in cancer^19^. Our understanding of the mechanism(s) of activation and alternative promoters’ impact on gene expression is unknown.

In this study, we analyze 100 CAGE-seq libraries from HCC patients and annotate 4083 (including 1031 novel) alternative promoters, representing 2926 multi-promoter (MP) genes, which are supported by histone modifications, Assay for Transposase-Accessible Chromatin sequencing (ATAC-seq), RNA Pol2 ChiP, and DNA methylation across patient cohorts and HepG2 cells. Transcription of alternative promoters is dominant outside CpG islands (CGIs), enriched for genes important in hepatocarcinogenesis, and their activation frequently results in the downregulation of the reference promoter. We show that CG-poor regions preferentially lose DNA methylation in tumor tissues, followed by the accessibility of the chromatin and Pol2 binding, resulting in alternative transcription from CG-poor regions. Collectively, our study elucidates the mechanism of activation and preferential underlying DNA sequence for alternative promoter activation in cancer.

## Results

### Widespread transcription of alternative promoters in HCC

**Fig. 1:**
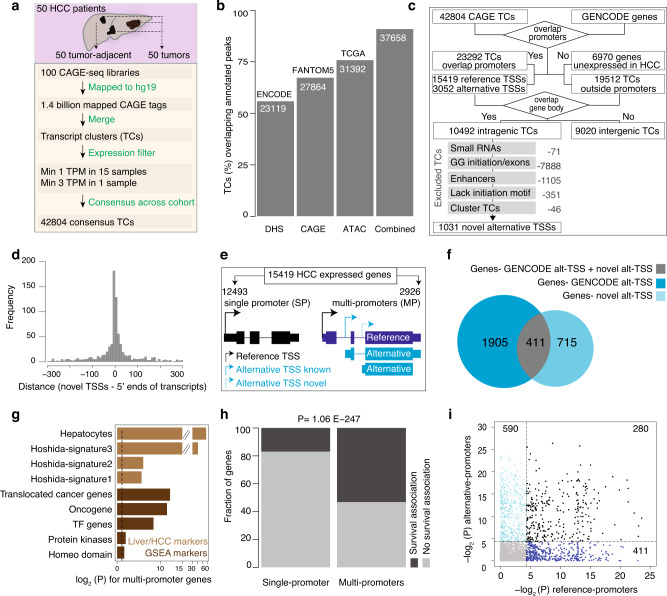
Annotation of alternative promoters in hepatocellular carcinoma (HCC) patients. **a** A schematic workflow to describe the mapping of CAGE-seq reads to define consensus transcript clusters (TCs) across the cohort. **b** Barplot shows the overlap of HCC TCs with the annotated FANTOM5 CAGE peaks and open chromatin peaks from ENCODE and TCGA. **c** A schematic workflow to annotate intragenic CAGE TCs as high-confidence alternative promoters. The workflow includes multiple filtering steps to exclude TCs that lack promoter features. **d** Distance between 5′ ends of novel TSSs and 5′ ends of RNA-seq and EST transcripts. **e** Classification of expressed genes into single promoter (SP) and multi-promoter (MP) genes based on the number of promoters. The promoter with the highest expression level (represented by arrow height) is assigned as the reference promoter. **f** Venn diagram shows the intersection of novel alternative promoters with known MP genes. **g** Enrichment of signature genes in MP genes compared to SP genes. *P* values were computed using a two-tailed Fisher’s exact test. **h** Distribution of survival-associated genes with SP and MP genes. The MP genes were significantly associated (*P* = 1.06E−247; Fisher’s exact test) with survival outcome. **i** The scatter plot shows the association of overall survival for reference and alternative promoters. *P* values were computed using the chi-squared test.

To determine the extent of alternative promoter usage in human HCC, we analyzed CAGE-seq data from 50 tumors and 50 matched tumor-adjacent normal liver tissues^8^ (Supplementary Table 1). We identified the 5′-end of CAGE TSSs (CTSSs) and quantified expression levels in tags per million (TPM). Proximal CTSSs within 20 nucleotides on the same strand were clustered to define transcript clusters (TCs) (Fig.1a and Supplementary Fig. 1a). We retained TCs expressed above 1 TPM in at least 15 samples and a minimum of 3 TPM in at least one sample, which resulted in 42,804 high-confidence consensus TCs expressed across the patient cohort (Fig. 1a and Supplementary Table 2). A majority of TCs (90%) were supported by FANTOM5 CAGE peaks^17^ and/or open chromatin regions from ENCODE^22^ and TCGA^7^ (Fig. 1b). Based on GENCODE transcript models, we identified promoters for 15419 expressed genes and alternative TSSs for 3052 annotated alternative transcripts. A significant fraction (10,492; 24.5%) of CAGE TCs were in intragenic regions (Fig. 1c), indicating these TCs are putative alternative promoters. We filtered intragenic CAGE TCs that represent drosha processing of pre-miRNAs^23^, snoRNAs 5′-ends capping^17^, exons post-transcriptional processing^11,12^, enhancer RNAs^24^, and those lacking a transcription initiator^15^ (Fig. 1c). The remaining TCs within 300 bases were clustered, resulting in 1031 novel alternative TSSs (Supplementary Table 3). A majority of the novel TSSs were supported by spliced transcripts (see methods) from RNA-seq^25,26^ and expressed sequence tag^27^ (Fig. 1d; Supplementary Fig. 1b). In total, we identified 4083 alternative TSSs in HCC (represented by 3052 annotated TSSs and 1031 novel TSSs) (Fig. 1c).

Based on the number of promoters, we classified the 15,419 expressed genes into either single-promoter (SP) (12,493; 80.3%) or MP (2926; 19.7%) genes (Fig. 1e, Supplementary Table 3 and Supplementary Table 4). Promoters with the highest mean expression level across the cohort were assigned as the reference (major) promoters, whereas the remaining were assigned as alternative (minor) promoters (Fig. 1e, see methods). Novel alternative TSSs preferentially occur in genes without active alternative promoters (Fig. 1f), while others occurred in existing MP genes, resulting in genes having multiple alternative promoters (Supplementary Fig. 1c). Overall, 65% of alternative promoters are located downstream of the reference promoter (Fig. 1g), and 35% are upstream, emphasizing that purely assigning the most upstream TSS as the reference promoter is suboptimal. Two-thirds of the alternative promoters were shown to utilize a different N terminus or were located downstream of the N terminus of the protein (Supplementary Fig. 1d; Supplementary Table 3), as exemplified for *CDKN2A* and *ERBB2* (Supplementary Fig. 1e, f). MP genes were associated with diverse functions enriched in metabolic processes, signaling pathways, apoptosis, regulation of cell migration, and programmed cell death (Supplementary Fig. 1g and Supplementary Table 5). In contrast, SP genes were over-represented in translation, DNA repair, gene expression, and mRNA splicing. Notably, hepatocytic markers^28^, HCC signature genes^29^, and cancer-associated gene families (oncogenes, transcription factors, and protein kinases) from GSEA^30^ were overrepresented in MP genes (Fig. 1g). Clinically, overall survival (OS) is significantly overrepresented (*p* = 3.2E−78; Fisher’s exact test) by MP genes (Fig. 1h and Supplementary Table 3). This includes both genes with known or unknown roles in HCC and genes not previously described to rely on promoter switching, such as *SULF2* (Supplementary Fig. 1h). Patients’ outcome relative to MP genes was significantly associated (*P* = 5.2E−24; Fisher’s exact test) with the expression of alternative promoters (Fig. 1i). Alternative promoters associated with survival had shorter OS time (*P* = 2.8E−8; Fisher’s exact test) (Supplementary Fig. 1i). Moreover, MP genes were enriched for sorafenib resistance-genes^31^ (Supplementary Fig. 1j). The observed enrichment at the cohort level was recapitulated for 55% of individual samples. Collectively, these data support that alternative promoters and their usage are important in hepatocarcinogenesis and for patient outcomes.

### Impact of alternative promoters on gene expression

**Fig. 2:**
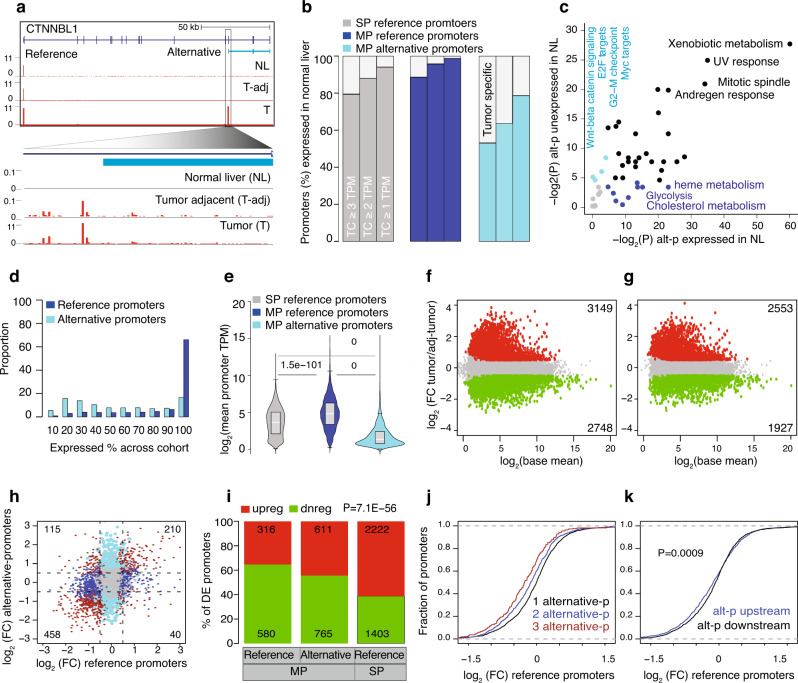
The impact of alternative promoters in gene expression regulation. **a** A UCSC browser screenshot of the *CTNNBL1* gene with CAGE tags across the normal liver, tumor-adjacent liver tissues, and HCCs. The alternative promoter is zoomed in to show the CTSS usage. **b** Percentage of the single promoter (SP) reference, multi-promoter (MP) reference, and alternative HCC promoters expressed across eight independent normal livers at different expression thresholds. HCC promoters are undetected in normal liver tissues and show tumor-specific activation of alternative promoters. **c** Enrichment of cancer hallmark terms associated with alternative promoters expressed in normal livers and those unexpressed in normal livers. *P*-values were computed using Fisher’s exact test and corrected for multiple testing. **d** Distribution of reference and alternative promoters expressed across the HCC cohort. The *x*-axis indicates the percentage at which a promoter is expressed across the HCC cohort. **e** The average expression level of reference (*n* = 2926) and alternative (*n* = 4083) promoters of multi-promoter (MP) genes and single promoter (SP) genes (*n* = 12,493). Boxplots show the 5th, 25th, 50th, 75th, and 95th percentiles, where the central line is the median. *P*-values were determined by two-tailed unpaired *t*-tests. **f**, **g** Volcano plots show differentially expressed promoters (**f**) and genes (**g**) between tumors and tumor-adjacent tissues. *P*-values were computed using the Wald test. The cut-off *P*-value of 0.05 was FDR-corrected. **h** Expression fold change for reference and alternative promoter pairs. **i** Barplot shows the fraction of differentially expressed promoters (from panel **f**) that are classified as either upregulated or downregulated in tumors compared to tumor-adjacent tissues. *P* value was computed using the chi-squared test. **j** The distribution of fold change of reference promoters based on the number of one or more alternative promoters. **k** The distribution of fold change of reference promoters based on the upstream or downstream position of alternative promoters relative to its reference promoter. The *P*-value was computed using the Kolmogorov–Smirnov test.

We sought to understand how alternative promoters control gene expression. We first analyzed the expression of HCC promoters across eight independent normal livers^8,17^ and observed some alternative promoters were undetected in the normal liver but already expressed in the matched tumor-adjacent liver (cirrhotic liver parenchyma) (Fig. 2a, Supplementary Fig. 2a and Supplementary Table 4). At the expression threshold of a minimum of 3 TPM in at least 1 normal liver sample, approximately 50% of the alternative promoters were unexpressed in the normal liver compared to 15% of reference promoters (Fig. 2b). We lowered the expression threshold and observed a higher fraction of alternative promoters were undetected in the normal livers. MP genes with alternative promoters expressed in normal livers were enriched for metabolic-related pathways, reflecting tissue-intrinsic biology. On the contrary, tumor-specific alternative promoter genes were enriched in oncogenic pathways such as WNT/beta-catenin signaling, E2F, and Myc targets (Fig. 2c and Supplementary Table 6).

We next binned reference promoters based on decreasing expression levels and observed that alternative promoters were expressed at similar levels across corresponding bins (Supplementary Fig. 2b). To understand the expression dynamics of alternative promoters, we computed their extent of expression variability across the patient cohort. The reference promoters (irrespective of SP or MP genes) were constitutively expressed in all samples, while alternative promoters were expressed either constitutively or only in a subset of patients (Fig. 2d). Notably, alternative promoters unexpressed in normal livers were generally expressed in a subset of tumor tissues (Supplementary Fig. 2c). Although the mean expression levels of alternative promoters were lower than the matched reference promoters (Fig. 2e), some alternative promoters have a higher expression at the individual patient level, as highlighted in *CTNNBL1* (Supplementary Fig. 2a). In total, 1489 (36.4%) alternative promoters’ expression was higher than reference promoters in one or more tumor-adjacent samples, which further increased to 1716 (42%) alternative promoters among tumors (Supplementary Fig. 2d). This reflects a high variability in the expression level of alternative promoters across the cohort (Supplementary Fig. 2e).

We used DESeq2^32^ and identified 5223 promoters and 4480 genes significantly and differentially expressed (*P*-adjusted <0.05 and fold-change log_2_(absolute(0.5)) between tumor and tumor-adjacent tissues (Fig. 2f, g and Supplementary Table 7). Differentially expressed promoters overlapped in 99% with the differentially expressed genes (Supplementary Fig. 2f). The fold-change among differentially expressed promoters was higher in alternative promoters (Supplementary Fig. 2g), which were distributed predominantly among the higher expression tier (Supplementary Fig. 2h). Detection of a lower number of differentially expressed genes suggests that reference and alternative promoters might have an inverse expression pattern. As such, we observed a general trend of downregulation among reference promoters (Fig. 2h), whereas the alternative promoters were significantly (*P* = 0.00012; Fisher’s exact test) upregulated (Supplementary Fig. 2i). Alternative upstream promoters^33^ and downstream intragenic promoters^34^ can attenuate host gene expression. Thus we asked whether genes downregulated in HCC are enriched among MP genes. The majority of differentially downregulated promoters are MP genes, while SP genes were upregulated (Fig. 2i), which held true across different functional classifications of genes except for KEGG signaling pathways (Supplementary Fig. 2j). Consistent with this observation, we showed that the reference promoters were downregulated when one or more alternative promoters are located upstream (Fig. 2j, k), which may interfere with the elongating polymerase leading to transcript downregulation. Collectively, this highlights that the transcription of many alternative promoters in tumors affects key cancer-related pathways and leads to the downregulation of the reference promoter.

### Alternative promoters have distinct underlying DNA sequences and promoter architecture

**Fig. 3:**
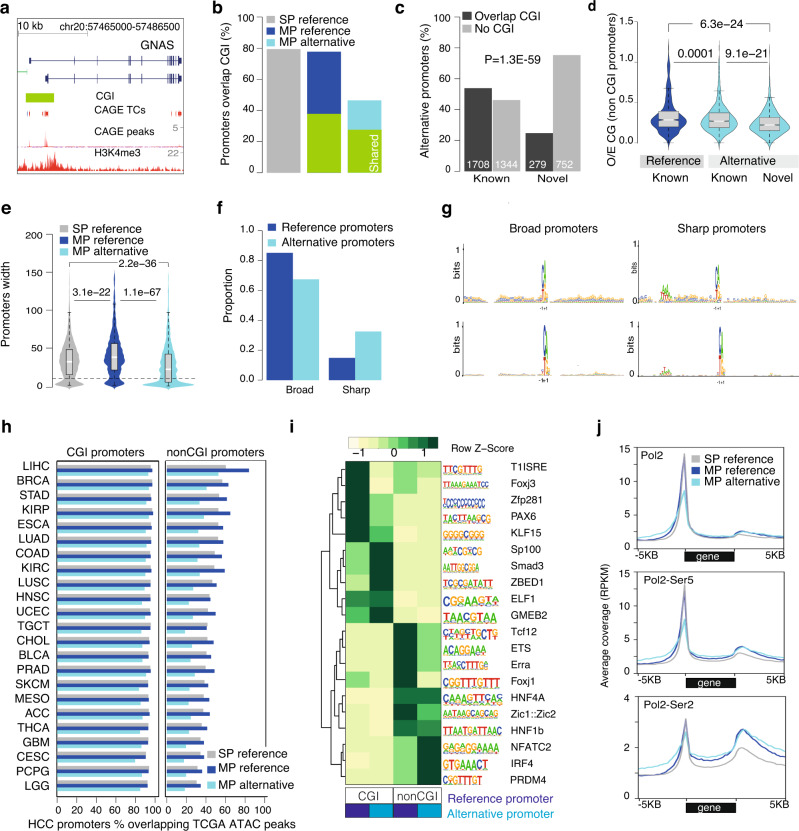
Promoter architecture of reference and alternative promoters. **a** A UCSC browser screenshot of *GNAS* gene along with CpG island (CGI), CAGE-seq, and H3K4me3 tracks. The reference and alternative promoters have a shared CGI. **b** Number of promoters overlapping CGIs across single promoter (SP) reference (*n* = 9997), multi-promoter (MP) reference (*n* = 2290), and MP alternative (*n* = 1987) promoters. CGIs shared by reference (*n* = 1050) and alternative (*n* = 1170) promoters are highlighted in green. **c** Proportion of annotated and novel alternative promoters overlapping CGIs. *P*-value was determined using Fisher’s exact test. **d** Distribution of CG density across a known reference (*n* = 776), known alternative (*n* = 1481), and novel (*n* = 1387) nonCGI promoters. *P*-values were determined by two-tailed unpaired *t*-tests. Boxplots show the 5th, 25th, 50th, 75th, and 95th percentiles, where the central line is the median. **e** Distribution of promoter width of SP reference (*n* = 12,493), MP reference (*n* = 2926), and alternative (*n* = 4083) promoters. *P*-values were determined by two-tailed unpaired *t*-tests. Boxplots show the 5th, 25th, 50th, 75th, and 95th percentiles, where the center line is the median. **f** Barplot shows alternative promoters have a higher proportion of sharp promoter shape relative to reference promoters. **g** Sequence motifs around TSSs of sharp and broad promoters for reference and alternative promoters. **h** Barplot shows the fraction of HCC promoters that overlapped with TCGA pan-cancer ATAC-seq peaks. Promoters were classified into two groups based on their overlap with CGIs. **i** Heatmap shows transcription factor motifs enriched across reference and alternative promoters as well as their overlap with CGIs. **j** The average coverage of RNA polymerase II (Pol2), phosphorylation modification at serine 5 (Pol2-Ser5; initiation of Pol2) and serine 2 (Pol2-Ser2; elongation by Pol2) on the large subunit of Pol2 in HepG2 cells across the gene body.

Two-thirds of human genes have CGIs within their promoters^35^, as exemplified for *GNAS* (Fig. 3a), *ERBB2*, and *COMT* (Supplementary Fig. 3a, b). In addition, thousands of genes have CGIs in their gene bodies (intragenic CGIs) that can act as novel alternative promoters during development^36^. We sought to understand whether intragenic CGIs (CG-rich regions) act as tumor-specific alternative promoter regions in HCC. Compared to 80% of reference promoters, only 48.6% (1987 out of 4083) of alternative promoters overlapped with CGIs (Fig. 3b), which is consistent with the depletion of CGIs among annotated alternative promoters^37–39^. A significant fraction of these alternative promoter CGIs (1170 out of 1987) was also shared with reference promoters (Fig. 3a, b). While CGIs are depleted among annotated alternative promoters^37–39^, CGIs were significantly further depleted (*P* = 1.9E−61; Fisher’s exact test) among novel alternative promoters (Fig. 3c). Alternative promoters overlapping CGIs had a lower observed to expected (O/E) ratio of CG dinucleotides (Supplementary Fig. 3c) and shorter CGIs lengths (Supplementary Fig. 3d). Notably, even among the nonCGI alternative promoters, the O/E CG ratio was significantly (*P* = 9.1E−21; *t*-test) lower in novel alternative promoters (Fig. 3d), which may emphasize a preferential activation of alternative promoters from CG-poor regions.

As such, we hypothesized that to adapt to the evolving cancer transcriptome, alternative promoters might be distinctly regulated, and this regulation might be mediated via a difference in their promoter architecture, chromatin accessibility, transcription factor association, and RNA Pol2 dynamics. CAGE TCs overlapping reference promoters have significantly broader widths (Fig. 3e, f), which are termed broad promoters^11^, and characterized by multiple initiation sites with a TC (Supplementary Fig. 3e). Contrary, CAGE TCs overlapping alternative promoters have narrow widths (Fig. 3e, f), which are termed sharp promoters^11^ and have one dominant TSS (Supplementary Fig. 3f). Both promoters have a well-defined transcription initiation motif, while the TATA box was positionally constrained upstream of sharp promoters (Fig. 3g), as previously observed^11,12^. The measure of chromatin accessibility of HCC promoters across TCGA ATAC-seq peaks^7^ revealed that most of the reference and alternative CGI promoters were uniformly accessible across cancers (Fig. 3h). While chromatin accessibility of nonCGI promoters varied across tumor types (Fig. 3h), reflecting CG-poor alternative promoters have tissue-specific accessibility and expression. The frequency of ENCODE transcription factor ChIP-seq peaks was significantly higher (*P* = 0.026; *t*-test) in reference promoters compared to its alternative promoters (Supplementary Fig. 3g). De novo analysis of transcription factors revealed that reference and alternative nonCGI promoters were enriched for liver-specific transcription factors (Supplementary Table 8) such as hepatocyte nuclear factor 1A (HNF1A) and HNF4A (Fig. 3i; Supplementary Fig. 3h), which is consistent with previously observed tissue-specific transcription factor enrichment among nonCGI promoters^40^. Notably, the transcription factor NFATC2 was enriched only on nonCGI alternative promoters. Recently, hepatic NFAT signaling was reported to regulate inflammatory cytokine expression in cholestasis^41^. Lastly, RNA polymerase II (Pol2) was enriched at similar levels in both SP and MP genes while initiating Pol2 modified by phosphorylation of serine 5 (Pol2-Ser5) and elongating Pol2 modified by phosphorylation of serine 2 (Pol2-Ser2) on its carboxy-terminal domain (CTD) were higher among MP genes (Fig. 2j). These data were validated by GRO-seq (Supplementary Fig. 3i, j). Thus, alternative promoters have distinct regulatory architecture characterized by low CG content, sharp promoter shape, and enriched for liver tissue-specific transcription factors.

### Alternative promoters have distinct chromatin architecture

**Fig. 4:**
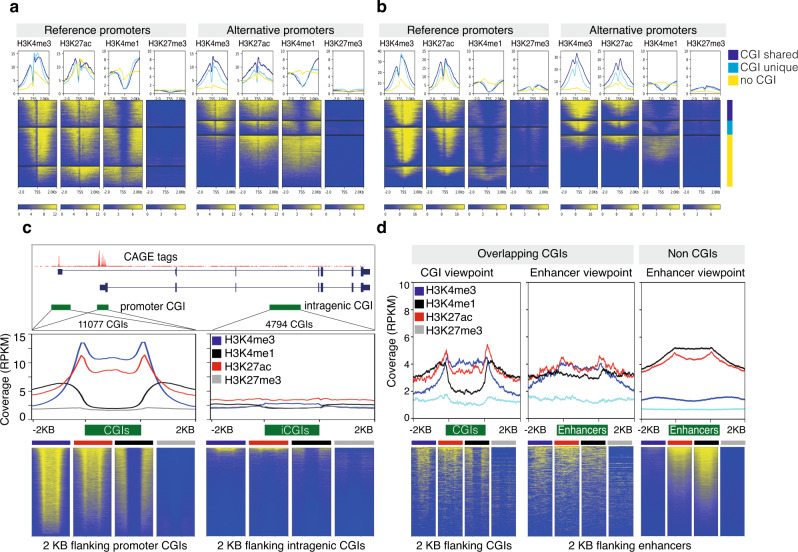
The landscape of histone modifications around the reference and alternative promoters. **a** Line plots show average histone modifications (H3K4me3, H3K27ac, H3K4me1, H3K27me3) levels of four HCC patients around reference (left panel) and alternative (right panel) promoters. Promoters are classified into three groups based on their overlap with CpG islands (CGIs). CGIs shared by reference and alternative promoters are classified as shared CGI. Heatmaps on the bottom show histone modifications for each promoter across three groups. **b** Enrichment of histone modifications (H3K4me3, H3K27ac, H3K4me1, H3K27me3) data in HepG2 cells, similar to that of the panel (**a**). **c** A UCSC browser screenshot of reference and alternative promoters overlapping promoter CGIs along with an intragenic CGI lacking CAGE tags. Line plots in the middle show the average coverage of histone marks (H3K4me3, H3K27ac, H3K4me1, H3K27me3) along promoter CGIs (left) and intragenic CGIs (right). CGIs of varying lengths are scaled between start and end. Heatmaps on the bottom show histone signals for CGIs. **d** Enrichment of histone modifications (H3K4me1, H3K4me3, H3K27ac, H3K27me3) on HepG2 enhancers overlapping CGIs (*n* = 791) and non-overlapping CGIs (*n* = 20891). CGIs and enhancers of varying lengths are scaled between start and end. Heatmaps on the bottom show histone signals for individual CGIs and enhancers.

A majority of MP genes have both CG-rich and CG-poor promoters. Thus, we asked whether the distinct chromatin architecture associated with the CG content in promoters^42,43^ can coexist within a gene. We aligned H3K4me1, H3K4me3, H3K27ac, and H3K27me3 modifications along dominant TSSs that were grouped based on their overlap with CGIs. Reference and alternative CGI promoters have divergent H3K4me3 and H3K27ac marks around the nucleosome-depleted region (NDR) at TSSs both in HCC patients^6^ (Fig. 4a) and HepG2 cells (Fig. 4b). Divergent H3K4me1 peaks were located further away from TSSs (Fig. 4a, b), which flanked CGIs and H3K4me3 boundaries^44–46^ (Fig. 4c). On the other hand, reference and alternative nonCGI promoters had low levels of H3K4me3 asymmetrically deposited downstream of TSSs, while H3K4me1 marks were non-divergent and enriched only at TSSs. H3K4me3 marks are influenced by CG density^47^. Thus H3K4me3 peaks are broader in CG-rich promoters (Supplementary Fig. 4a). As the width of H3K4me3 peaks becomes narrow in CG-poor promoters, the distance between divergent H3K4me1 peaks becomes shorter and appears continuous. Compared to nonCGI reference promoters, nonCGI alternative promoters have low levels of H3K4me3 (Fig. 4a) and lower CG density (Supplementary Fig. 4b). The observed histone modifications at nonCGI alternative promoters resemble epigenetic features of enhancers, as previously observed^48^. While both reference and alternative promoters overlap with ChromHMM HepG2 enhancers (Supplementary Fig. 4c), we sought to understand whether the low CG density of alternative promoters can explain the observed histone modification as enhancers. To this end, we classified ChromHMM HepG2 enhancers (excluding promoter regions) into 791 CGI enhancers and 20,891 nonCGI enhancers (see “Methods”). Enhancers overlapping CGIs had similar patterns of H3K4me3 and H3K4me1 to that of CG-rich promoters (Fig. 4d). Similarly, nonCGI enhancers had low levels of H3K4me3 and high levels of H3K4me1 peaks (Fig. 4d) like that of nonCGI promoters. This reinforces the premise that H3K4me3 is linked to CG density^47^, and depletion of H3K4me3 on nonCGI promoters and enhancers is due to low CG density.

To understand why alternative transcription does not occur from intragenic CGIs, we analyzed H3K4me3, H3K4me1, and H3K27ac modifications and observed no enrichment (Fig. 4c), thus explaining the absence of transcription. Notably, divergent H3K27ac peaks were enriched on both CG-rich and CG-poor promoters, while they lacked H3K27me3 as expected in active genes (Fig. 4a, b). Furthermore, CGI promoters were enriched for H2A.Z, have a well-defined NDR, and (phased +1) downstream the nucleosome, whereas nonCGI promotes have undefined NDR (Supplementary Fig. 4d). In case of alternative CGI promoters, the gene body histone (H3K79me2, H3K36me3, and H4K20me1) marks presented NDR, while histones were continuous in nonCGI alternative promoters (Supplementary Fig. 4e). In conclusion, reference and alternative promoters have distinct chromatin architecture that is influenced by genomic CG density, thus revealing that two distinct chromatin architectures coexist in MP genes.

### Dynamic DNA (de)methylation landscapes around alternative promoters

**Fig. 5:**
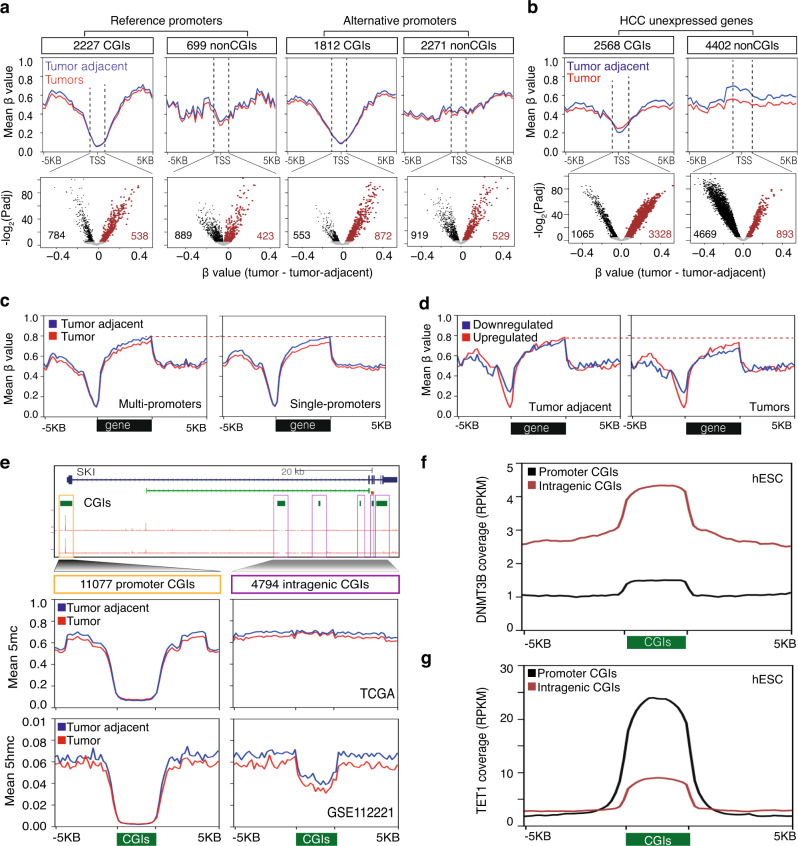
CG density influences DNA methylation landscapes. **a** Mean methylation levels (*β* values, top) around transcription start sites (TSSs) of reference and alternative promoters across the TCGA HCC cohort. Mean methylation levels of the TCGA HCC cohort are derived from 379 tumors and 51 tumor-adjacent tissues. Promoters overlapping with CpG islands (CGI) were separated from non-overlapping promoters. The scatter plots (bottom panel) show differentially hypermethylated (brown) and hypomethylated (black) CpGs in 500 nucleotides window around TSSs. *P*-values were determined by two-tailed unpaired *t*-tests between HCC tumors and tumor-adjacent tissues. *P* values were adjusted for multiple testing. **b** Mean methylation levels around TSSs of unexpressed genes across the TCGA HCC cohort. The scatter plots (bottom panel) show differentially hypermethylated (brown) and hypomethylated (black) CpGs in 500 nucleotides window around TSSs. *P*-values were determined by two-tailed unpaired *t*-tests between HCC tumors and tumor-adjacent tissues. *P* values were adjusted for multiple testing. **c** Mean methylation levels across TCGA tumors and tumor-adjacent tissues along gene bodies of multi-promoter (left panel) and single-promoter (right panel) genes. **d** Mean methylation levels along gene bodies of up/downregulated genes in tumor-adjacent tissues (left panel) and tumor tissues (right panel). **e** A UCSC browser screenshot showing promoter CGI, intragenic CGIs, and CAGE tags for the *SKI* gene. Zoomed view (top panels) shows the average methylation level of promoter CGIs, intragenic CGIs, and their flanking regions. CGIs of varying lengths are scaled between start and end. Zoomed view (bottom panels) shows the average demethylation (5hmC) levels. Mean methylation levels of the TCGA HCC cohort are derived from 379 tumors and 51 tumor-adjacent tissues. Mean methylation levels of the GSE112221 cohort are derived from 4 tumors and 4 tumor-adjacent tissues. **f** Coverage of DNMT3B binding on promoter CGIs and intragenic CGIs across human ES cells. **g** Coverage of TET1 binding on promoter and intragenic CGIs across human ES cells.

The CpGs in CG-poor regions are generally methylated, while CpGs within CGIs remain unmethylated in normal cells^49^ and aberrantly hypermethylated in cancer^2,50^. We sought to understand whether DNA methylation facilitates the transcription of alternative promoters in CG-poor regions. Promoters overlapping CGIs have low methylation levels at TSSs compared to nonCGI promoters (Fig. 5a). DNA methylation at TSSs decreased based on increasing CG density in CGI and nonCGI promoters (Supplementary Fig. 5a), which was more evident among nonCGI promoters. These findings were further validated using reduced representation bisulfite sequencing (RRBS) of HepG2 cells (Supplementary Fig. 5b). H2A.Z marks are mutually antagonistic with DNA methylation^51^, which, together with an observed H2A.Z enrichment on CGI promoters (Fig. 4d) provides an explanation for the depletion of methylation on CGI promoters. We next analyzed CG probes (±500 bases around TSSs) and identified hundreds of differentially hypo- and hypermethylated promoters (Fig. 5a, b), which significantly overlapped with the differentially expressed promoters (Supplementary Fig. 5c). Aberrant hypermethylation of CGI promoters was not globally observed among expressed genes, while it was prevalent among CGI promoters of unexpressed genes (Fig. 5b) as recently observed in colorectal cancer^52^. Regions with higher CpG density were more hypermethylated, while CpGs in nonCGI promoters were more hypomethylated (Supplementary Fig. 5c). This demonstrates that DNA methylation levels are influenced by the genomic CpG density at both expressed and unexpressed genes.

To understand why transcription of alternative promoters was depleted in intragenic CGIs (Fig. 3c and Fig. 4c), we sought to understand how the CpG density influences intragenic DNA methylation in tumors. Gene bodies have high levels of DNA methylation^20,53^, which we observed across both MP and SP genes (Fig. 5c). While gene body methylation was globally decreased in HCC tumors compared to the tumor-adjacent tissues (Fig. 5c), it was more evident among downregulated promoters (Fig. 5d). Notably, genes with intragenic CGIs had higher methylation levels in both tumors and tumor-adjacent tissues (Supplementary Fig. 5f), reflecting that the local CG density influences intragenic (de)methylation in tumors. High methylation levels of intragenic CGIs are opposite to that of promoter CGIs (Fig. 5e; Supplementary Fig. 5g). High levels of DNA methylation on intragenic CGIs remained globally unchanged in HCC tumors (Fig. 5e) while flanking regions had decreased methylation. As *DNMT3B* regulates intragenic DNA methylation levels^54^, high levels of DNA methylation levels are consistent with high levels of *DNMT3B* binding on intragenic CGIs (Fig. 5f). Intragenic CGIs have low levels of DNA demethylase (5hmC levels) activity (Fig. 5e) measured by TET-assisted reduced RRBS (TAB-RRBS)^6^, which is consistent with low levels of TET1 binding (Fig. 5g). Thus, the lack of active demethylation on intragenic CGIs may explain its sustained high level of methylation to maintain the repressive state, resulting in lack of activation of alternative promoters from intragenic CGIs (Fig. 4d). In contrast, alternative promoters with CG-poor regions in tumor tissues are sensitive to demethylation, which in turn may facilitate an open chromatin structure and transcription initiation.

### Deregulation of SETD2, H3K36me3, and DNA methylation facilitates open chromatin

**Fig. 6:**
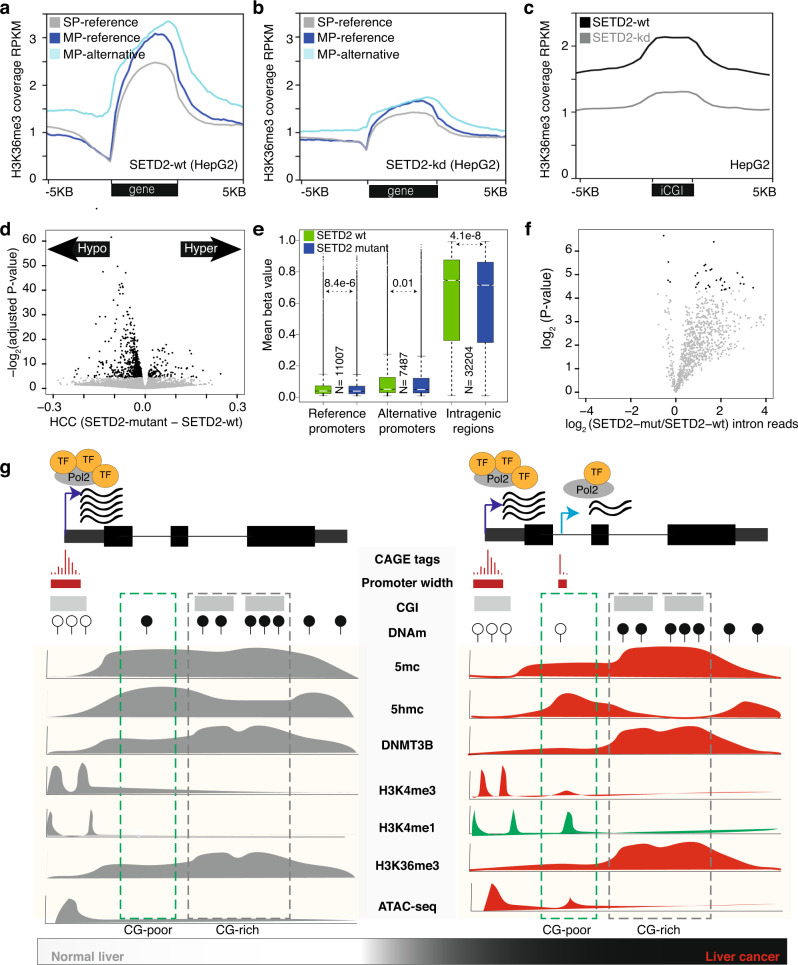
Regulation of SETD2. **a**, **b** Average coverage of H3K36me3 along gene body and flanking regions of the single-promoter (SP) and multi-promoter (MP) genes in SETD2-wt (*n* = 2, replicates merged) and SETD2-kd (*n* = 2, replicates merged) in HepG2 cells. **c** The average coverage of H3K36me3 along intragenic CGIs and flanking regions in SETD2-wt and SETD2-kd in HepG2 cells. **d** Volcano plot shows hypermethylated and hypomethylated CpGs between SETD2-mutant (*n* = 15) and SETD2 wild-type (*n* = 362) tumors from TCGA HCC patients. *P*-values were determined by two-tailed unpaired *t*-tests between SETD2-mutant and SETD2 wild-type groups. *P* values were adjusted for multiple testing. Y axis indicates the negative log2 value of adjusted *P* values. **e** Boxplots show average DNA methylation levels (beta values) of CpGs around reference promoters, alternative promoters, and intragenic regions. Mean methylation levels are derived from 15 SETD2-mutant and 362 SETD2 wild-type TCGA HCC patients. Boxplots show the 5th, 25th, 50th, 75th, and 95th percentiles, where the center line is the median. *P*-values were determined by two-tailed unpaired *t*-tests. **f** Volcano plot shows fold-change of intronic reads in SETD2-mutant (*n* = 15) versus SETD2 wild type (*n* = 362). *P*-values were determined by a two-tailed unpaired *t*-test. **g** Schematic representation to illustrate tumor-specific transcription of alternative promoters from CG-poor regions. The chromatin structure of intragenic CG-rich and CG-poor regions have different distributions of 5mC, 5hmC, H3K36me3, and DNMT3B, leading to the pervasive initiation of alternative promoters from CG-poor regions.

The methyltransferase *SETD2* deposits H3K36me3^55^ after Pol2 passage^56^, which in turn recruits DNMT3B^21^ to maintain a repressive chromatin state^57^. Old yeast cells have decreased H3K36me3 levels and increased intragenic transcripts^58^, while DNA methylation decreases with age in humans^59^. DNA methylation is globally reduced within gene bodies (Fig. 5c); thus, we reasoned that impaired *SETD2* might accelerate the loss of the repressive chromatin state, facilitating alternative transcription. Knockdown of *SETD2* globally decreased H3K36me3 signals from gene bodies (Fig. 6a) with relatively higher retention in CG-rich regions (Fig. 6b). Compared to normal hepatocytes (HepaRG), HepG2 cells had lower H3K36me3 marks (Supplementary Fig. 6a, “Methods”), suggesting a loss of H3K36me3 in tumor cells. As H3K36me3 recruits DNMT3B^21^, the gene body coverage of H3K36me3 (Supplementary Fig. 6b) coincides with the DNMT3B coverage (Fig. 6c). Using CRISPR epitope tagging (through insertion) of DNMT3B^60^ revealed DNMT3B signals decreased along the gene body (Supplementary Fig. 5c), indicating a loss of the repressive epigenetic marks along the gene body.

We reasoned that if *SETD2* is mutated, this would lower H3K36me3 levels and result in reduced DNMT3B binding, increasing the loss of DNA methylation (hypomethylation) across gene bodies. To this end, we compared the DNA methylation landscapes of *SETD2* mutated HCC patients (*n* = 15) with *SETD2* wild-type HCC patients (*n* = 362). We observed that SETD2-mutant tumors were significantly hypomethylated and that demethylated CpGs were enriched in gene bodies overlapping CGIs (Fig. 6d, e and Supplementary Fig. 6d, e). Since CG-rich regions are marked by higher levels of H3K36me3 (via SETD2), *SETD2* mutations largely cause hypomethylation in CG-rich regions. Notably, SETD2-mutants have a high level of intronic reads (Fig. 6f), providing evidence for transcribed reads from introns. Predominant hypomethylation observed across gene bodies in tumors was more prevalent in SETD2-mutants, as *SETD2* mutations accelerate the epigenetic control, compromising the maintenance of DNA methylation and resulting in activation of alternative promoters. Thus, the interplay of DNA methylation, chromatin accessibility, and histone modification in cancer preferentially facilitates alternative transcription in CG-poor regions (Fig. 6g).

## Discussion

Alternative promoters in human HCC were annotated using high-resolution CAGE-seq. Within this patient cohort, we annotated a total of 4083 (3052 annotated and 1031 novel) alternative promoters. Since there is no consensus on which promoter should be assigned as a reference or alternative, a highly expressed promoter is often annotated as the reference promoter^16,19^, which is what we used in this study. Few alternative promoters have expression levels similar to their reference promoters, and in these cases, the annotation of reference and alternative promoters is interchangeable across cohorts/studies. In this study, we analyzed tumor samples and ensured that the reference promoter was expressed in the normal liver; thus, tumor-specific promoters are annotated as alternatives. Besides, our annotation of alternative promoters is supported by multiple data sets across HCC patient cohorts and in HepG2 liver cancer cells, thus providing a framework for future functional studies.

We found that CG density is the major distinguishing feature between reference (high CG density) and alternative (low CG density) promoters. Depletion of HCC alternative promoters from intragenic CGIs is notably different than alternative promoters usage from intragenic CGIs during developmental stages^36^ or gene upstream CGIs^61^ in adult tissues. Human promoters are mostly CG-rich^35,62^ and have distinct promoter architecture and regulation^11^ compared to CG-poor promoters which are mostly tissue-specific^17^. Accordingly, a majority of HCC nonCGI alternative promoters are detected in lower proportion across other tumor types (Fig. 3h), indicating they drive tissue-specific or cell type-specific transcription and are enriched for liver-specific transcription factors. This highlighted the unexpected presence of two distinct regulatory promoter architectures within a gene that is widespread in cancer. While reference promoters are generally ubiquitous and represent the predominant degree of transcription, it is important to understand that cell type-specific transcription might contribute to tissue-specific alternative promoters. This requires implementation of single-cell capturing the 5′-ends^63^, while single-cell methods currently in use are enriched toward genes at the 3′-ends and, therefore, insensitive for promoter detection. Single-cell capturing 5′-ends in mouse neurons^63^ has revealed many alternative promoters detected in bulk tissues were detected in a single cell, which suggests that most alternative promoters might be co-expressed in the same cell.

We also found that epigenetic (histone modifications and DNA methylation) landscapes of reference and alternative promoters are dramatically different, which is due to their differences in CG density. The H3K4me3 marks are enriched on CGIs^47^, while we showed that intragenic CGIs lack H3K4me3 marks due to the retention of high DNA methylation in the tumor. It is known that aged cells (and cancer cells) generally lose DNA methylation from gene bodies^59^. We described a hierarchy that explained preferential loss or retention of intragenic DNA methylation dependent upon the flanking CG density where CG-rich regions retain high tumor DNA methylation. This preferential retention of intragenic DNA methylation is regulated by *SETD2*, H3K36me3, and DNMT3B as they preferentially bind to CG-rich regions. This leads to intragenic CG-rich regions with more repressive chromatin marks, and that would require higher levels of DNA demethylating enzyme to make their chromatin accessible. Instead, these intragenic CG-rich regions have low levels of *TET1* binding and, thus, have reduced active demethylation. Thus, intragenic CGIs are less favorable to act as alternative promoters in cancer, though they can act as alternative promoters during embryonic development and cell differentiation^36^. On the contrary, intragenic CG-poor regions have lower levels of repressive chromatin state and higher levels of *TET1* binding, which collectively makes their DNA sensitive to active demethylation and accessible chromatin to experience pervasive Pol2 binding^64^ that, upon the availability of a tissue-specific transcription factor will efficiently initiate and elongate these Pol2. We propose that other tumors have similar mechanisms in activating alternative promoters, while their preferred location will be dependent upon DNA demethylated sites that are enriched for tissue-specific transcription factors of the specific tumor tissue.

The general view on why some genes have multiple promoters is that a cell needs more expression of specific genes, and transcription from multiple promoters may add flexibility manifested through different molecular mechanisms in regulating gene expression^65^. However, alternative promoters are widespread in cancer, raising an interesting question as to whether it is advantageous for tumor cells. We discussed different scenarios where alternative promoters offer functional advantages for tumors. Firstly, alternative promoters can downregulate reference promoters through transcriptional interference, which often coincides with important genes, including hepatocyte-specific markers (Supplementary Fig. 2f). Accelerated loss of expression of these genes may help hepatocytes to lose their cellular identity and gain a new identity as the malignant tumor. To develop the cellular identity, tumor cells might utilize signaling pathways differently in a globally changed cancer background. As such, alternative promoters detected only in tumors were enriched for genes in signaling pathways (Fig. 2c), which globally did not downregulate reference promoters (Supplementary Fig. 2f). This suggests that signaling pathway genes co-opt both promoters, where tumor cells utilize signaling pathways differently via alternative promoter usage (for example in *CDKN2A* and *ERBB2* (Supplementary Fig. 1e, f)). From the clinical perspective, MP genes were significantly associated with OS, similar to recent observations in multiple myeloma^66^. This opens an avenue to elucidate improved diagnostic biomarkers for the early onset of cancer, as many alternative promoters are expressed in a tumor-specific manner. Signaling pathways genes are often potential targets in designing drugs. Hence future work is needed to understand whether targeting tumor-specific alternative transcripts might increase the drug specificity and efficacy. While our analyses suggest an important role of alternative promoters in liver cancer, their biological significance in the full spectrum of liver cancer remains unexplored. The selective use of alternative promoters often goes uncharacterized in gene expression analyses in standard RNA-seq analyses, and thus we encourage scientists to carefully inspect whether their genes of interest are under alternative promoter regulation.

## Methods

### Mapping of HCC CAGE-seq reads to define CTSSs

Raw CAGE-seq reads were downloaded from a previous study^8^. The CAGE-seq reads were mapped to the human genome (hg19) with bowtie2^67^ by allowing up to two mismatches. On average, around 80-90% of sequenced reads were mapped, resulting in an average of 15-16 million mapped reads (Supplementary Table 1). The 5′ end of mapped CAGE reads provides TSSs at single nucleotide resolution and is termed as CTSSs. Low-quality and multi-mapping CTSSs with MAPQ scores below 20 were filtered using SAMtools^68^. The CTSSs that overlapped with 421 blacklisted regions from ENCODE^69^ were excluded. The CAGE protocol often adds “G” at the 5′-ends of capped TSS^11^, which generally remains unmapped and shifts TSS by 1 nucleotide. We used SAMtools and detected mapped reads with unmapped “G” at the first base. We then corrected such CTSSs by shifting the TSS position by 1 nucleotide as described before^12^.

### TCs and generating consensus TCs across the cohort

To define TCs for each sample, we clustered CTSSs in the same strand that overlapped within 20 nucleotides^12^. The CTSSs with the highest expression level within TC were defined as the dominant CTSS. All CTSSs within the TC were added that defined the expression level of TC. We then computed the interquartile width of the TCs by trimming the edges of the TCs in the range of 0.1–0.9 percentile of the TC. To define the consensus TCs across the cohort, we clustered TCs from all patients and computed their expression levels. To ensure that consensus TCs have robust expression levels, we retained TCs only if their expression was higher than 1 TPM in at least 15 samples (15% of the cohort) and had a minimum of 3 TPM at least in 1 sample.

### Assignment of reference and alternative promoters among annotated transcripts

A gene with a single promoter was classified as a single promoter gene, and its promoter was assigned the reference (alias as a primary, major, main) promoter. Genes with two or more promoters were classified as MP genes. The promoter with the highest mean expression level at the population level, which is also expressed in normal liver tissue, was assigned as the reference promoter. The remaining promoters of that gene were assigned as alternative (alias minor) promoters of that gene. The terms reference TSS and alternative TSS have been used ambiguously.

### Annotation of novel alternative promoters

To annotate novel alternative promoters, we implemented a pipeline (Fig. 1c) that systematically filtered intragenic TCs that are unlikely to represent true promoters. We filtered CAGE TCs overlapping small RNAs, drosha processing site of pre-miRNAs^23^, and 5′ ends capping of snoRNAs^17^. Thousands of intragenic CAGE TCs are detected within exons that represent post-transcriptional processing and are characterized by GG-initiation^11,12^. We filtered intragenic CAGE TCs with GG-initiation and those overlapping with coding exons and annotated enhancers^24,70^. We also annotated novel enhancers from CAGE TCs by computing directionality^24^. Briefly, the directionality score (DS) for intragenic TCs was calculated by measuring the expression level of TCs in forward and reverse strands, where DS = (Forward − Reverse)/(Forward + Reverse). CAGE TCs with directionality scores between 0.5 and −0.5 were classified as enhancer RNAs. On the remaining TCs, we only retained those that have YR-initiation or YC-initiation initiation motif^15^. The remaining 1077 TCs represent high-confidence true promoter tags that act as alternative TSSs to annotated genes. We clustered proximal TCs with 300 bases, thus resulting in 1031 novel alternative TSSs. To provide evidence of RNA transcripts for these 1031 alternative TSSs, we analyzed transcript models from RNA-seq^25,26^ and expressed sequence tags^27^. We excluded transcripts that are proximal (within 300 bases apart) to 5′ ends of annotated Ensembl transcripts. The remaining transcripts with distinct 5′ ends overlapped within 300 bases of novel alternative TSSs.

### Histone profiles of annotated HepG2 enhancers

We downloaded previously annotated enhancers^24,70^ across different tissues and cell types, and ChromHMM^71^ annotated enhancers on HepG2 cells. We intersected ChromHMM HepG2 enhancers with other enhancers and ensured that enhancers were annotated across multiple datasets. We excluded enhancers that overlapped with gene promoters. The remaining 21682 enhancers were divided into two groups based on overlap with CGIs. Enhancers that overlapped (a minimum of 10% of enhancer length) with CGIs were annotated as CGI enhancers (*n* = 791). The remaining non-overlapping (*n* = 20891) enhancers were annotated as nonCGI enhancers.

### Mapping and visualization of histone ChIP-seq of HCC patients and HepG2 cells

We downloaded the raw sequence reads of H3K4me1, H3K4me3, H3K27ac, and H3K27me3 ChIP-seq data for four HCC patients^6^. We downloaded raw sequence reads of H3K36me3 marks on SETD2 wild type and SETD2 knockdown conditions on HepG2 cells^72^. The raw sequence reads were mapped using bowtie2^67^ and excluded multi-mapping reads.

### Analysis of DNA methylation of HCC patients and HepG2 cells

The Illumina 450 K DNA methylation data from the TCGA HCC cohort was downloaded from UCSC Xena hub^73^. The methylation levels were computed as beta values in the range of 0–1. The reduced RRBS of HepG2 cells was downloaded from ENCODE. The TET-assisted reduced RRBS (TAB-RRBS) of HCC patients was downloaded as a beta value^6^. For each CpG, we computed the average methylation levels across the cohort, separately for tumor and tumor-adjacent tissues. Differentially methylated CpGs around promoter regions were identified using a *t*-test on individual methylation levels between tumor and tumor-adjacent tissues. The *P*-value was adjusted for multiple corrections and *P*-value less than 0.05 was defined as significantly methylated CpGs. For visualization of average methylation levels around TSSs and CGIs, the mean beta value was converted into bigwig tracks and plotted average beta value using deepTools^74^.

For the analyses of DNA methylation landscapes induced by *SETD2* mutations, we separated TCGA HCC patients into two groups, namely, SETD2 mutants (*n* = 16) and *SETD2* wild type (n = 352). Differentially methylated CpGs were identified using a *t*-test on individual methylation levels between SETD2 mutants and SETD2 wild-type tumors. The *P*-value was adjusted for multiple corrections and a *P*-value less than 0.05 was defined as significantly methylated CpGs.

### Visualization of metaplots across genes and CGIs

All metaplots across genes and CGIs were plotted using deepTools^74^. From mapped BAM files, we first generated coverage as wig tracks which are normalized as RPKM using default parameters from deepTools. For the genes and CGIs metaplot, variable lengths of genes and CGIs were scaled between start and end.

### Enrichment of transcription factor and *de-novo* motif analysis

We downloaded transcription factors (TFs) ChIP-seq peaks for HepG2 from ENCODE^75^. To calculate the density of TFs, we intersected TFs peaks with promoter regions by using bedtools^76^. Different TFs overlapping promoters were summed to determine the total number of TFs per promoter. To identify overrepresented transcription factors, we performed motif analyses using HOMER^77^. We used 500 bases around TSSs as the search region to detect motifs. The background regions were controlled for nucleotide composition and selected by default by HOMER. We first identified motifs using the following parameter “findMotifsGenome.pl -chopify -len 8,10,12 -S 25 -size −500,500”. To compare these identified motifs with known motifs, we reran prediction using the following command “findMotifsGenome.pl -chopify -len 8,10,12 -S 25 -mcheck -mknown -size −500,500”. For visualization of motifs, we computed the matrix of p-value across promoter types and plotted them as heatmaps.

### Signature genes from the literature

Curated signature genes for hepatocytes^28^, Hoshida signature genes^29^, sorafenib resistance signature^31^, and GSEA cancer hallmark genes^30^ were downloaded from the literature. We are interested in these gene signatures with annotated SP and MP genes. We computed Fisher’s exact test to determine the statistical significance of the overlap between signature genes with single and MP genes.

### Patients’ OS analysis

To compute the OS of patients associated with expression level for each transcript, we downloaded TCGA LIHC clinical data^2,78^ and transcript expression levels from UCSC Xena hub^73^. Each transcript was sorted based on its expression levels and classified into high-expression and low-expression groups. We associated the expression levels of these transcripts from two (high-expression and low-expression) groups with the survival status of patients and performed Kaplan–Meier analyses. For MP genes, we performed Kaplan–Meier analysis on the reference transcript and on the alternative transcript.

### Differential expression at promoter and gene level

Differentially expressed genes and promoters were identified using DE-seq2^32^. The significance cut-off was defined at an adjusted *P*-value of 0.05 and log2 fold-change of absolute (0.5). For gene level analysis, we summed the expression levels of reference and alternatives promoter for each MP gene.

### Analysis of N-terminus of protein and UniProt domains

To compare whether alternative promoters altered the N-terminus of the protein, we analyzed only those alternative transcripts that have assigned UniProt protein domains and hence excluded noncoding transcripts and novel alternative promoters. We compared the N-terminus of the reference and alternative promoters, and if they had different start codons, they were assigned as different N-terminus proteins.

### Classification of CGIs and observed/expected CG ratio

Annotated CGIs were downloaded from the UCSC database^27^. We intersected CGIs with assigned promoter regions of annotated transcripts, and those overlapping promoters were assigned as promoter CGIs. The remaining CGIs that overlapped gene bodies were classified as intragenic CGIs. The remaining CGIs were classified as intergenic CGIs. The observed/expected (O/E) CG dinucleotides for a given genomic window were calculated using the following formula O/E CG = (CG count) (genomic window)/(C count * G count). We used “bedtools nuc” function from Bedtools^76^ to measure these values and computed the O/E CG ratio for each genomic window.

### Reporting summary

Further information on research design is available in the Nature Portfolio Reporting Summary linked to this article.

## Supplementary information

Supplementary Information

Reporting Summary

Supplementary Tables

Supplementary Table 2

## Data Availability

All sequencing data analyzed in this study are publicly available. Data accession codes are provided: HCC CAGE data (dbGap phs000885.v1.p1)^8^, normal liver CAGE data (DDBJ DRA: DRA000991: https://fantom.gsc.riken.jp/5/data/)^17^, HCC histone modifications and RRBS data (GEO GSE112221)^6^, H1ESC DNMT3B (GEO GSE150072)^79^ and HepG2 shCtrl and shSETD2 H3K36me3 data (GEO GSE110323)^72^. The data generated by the TCGA (https://www.cancer.gov/tcga)^2,7^ was downloaded from https://xenabrowser.net/^73^. ENCODE data^22,60^ was downloaded from https://www.encodeproject.org/. Processed data are provided as supplementary tables. Source data are provided in this paper.

## Source data

Source Data

## References

[1] Sung H (2021). Global Cancer Statistics 2020: GLOBOCAN estimates of incidence and mortality worldwide for 36 cancers in 185 countries. CA Cancer J. Clin..

[2] Cancer Genome Atlas Research Network. (2017). Comprehensive and integrative genomic characterization of hepatocellular carcinoma. Cell.

[3] Villanueva A (2015). DNA methylation-based prognosis and epidrivers in hepatocellular carcinoma. Hepatology.

[4] Schulze K (2015). Exome sequencing of hepatocellular carcinomas identifies new mutational signatures and potential therapeutic targets. Nat. Genet..

[5] Fujimoto A (2016). Whole-genome mutational landscape and characterization of noncoding and structural mutations in liver cancer. Nat. Genet..

[6] Hlady RA (2019). Integrating the epigenome to identify drivers of hepatocellular carcinoma. Hepatology.

[7] Corces, M. R. et al. The chromatin accessibility landscape of primary human cancers. *Science* **362**, 420–433 (2018). 10.1126/science.aav1898 PMC6408149 30361341

[8] Hashimoto K (2015). CAGE profiling of ncRNAs in hepatocellular carcinoma reveals widespread activation of retroviral LTR promoters in virus-induced tumors. Genome Res..

[9] Lin Z (2020). RNA m(6) A methylation regulates sorafenib resistance in liver cancer through FOXO3-mediated autophagy. EMBO J..

[10] Lenhard B, Sandelin A, Carninci P (2012). Metazoan promoters: emerging characteristics and insights into transcriptional regulation. Nat. Rev. Genet..

[11] Carninci P (2006). Genome-wide analysis of mammalian promoter architecture and evolution. Nat. Genet..

[12] Nepal C (2013). Dynamic regulation of the transcription initiation landscape at single nucleotide resolution during vertebrate embryogenesis. Genome Res..

[13] Kawaji H (2014). Comparison of CAGE and RNA-seq transcriptome profiling using clonally amplified and single-molecule next-generation sequencing. Genome Res..

[14] Haberle V (2014). Two independent transcription initiation codes overlap on vertebrate core promoters. Nature.

[15] Nepal C (2020). Dual-initiation promoters with intertwined canonical and TCT/TOP transcription start sites diversify transcript processing. Nat. Commun..

[16] Chia M (2021). High-resolution analysis of cell-state transitions in yeast suggests widespread transcriptional tuning by alternative starts. Genome Biol..

[17] Consortium F (2014). A promoter-level mammalian expression atlas. Nature.

[18] Baranasic, D. et al. Multiomic atlas with functional stratification and developmental dynamics of zebrafish cis-regulatory elements. *Nat. Genet*. **54**, 1037–1050 (2022). 10.1038/s41588-022-01089-w PMC9279159 35789323

[19] Demircioglu D (2019). A Pan-cancer transcriptome analysis reveals pervasive regulation through alternative promoters. Cell.

[20] Maunakea AK (2010). Conserved role of intragenic DNA methylation in regulating alternative promoters. Nature.

[21] Neri F (2017). Intragenic DNA methylation prevents spurious transcription initiation. Nature.

[22] Meuleman W (2020). Index and biological spectrum of human DNase I hypersensitive sites. Nature.

[23] Nepal C (2016). Transcriptional, post-transcriptional and chromatin-associated regulation of pri-miRNAs, pre-miRNAs and moRNAs. Nucleic Acids Res..

[24] Andersson R (2014). An atlas of active enhancers across human cell types and tissues. Nature.

[25] Hon CC (2017). An atlas of human long non-coding RNAs with accurate 5’ ends. Nature.

[26] Iyer MK (2015). The landscape of long noncoding RNAs in the human transcriptome. Nat. Genet..

[27] Lee BT (2022). The UCSC Genome Browser database: 2022 update. Nucleic Acids Res..

[28] Aran D, Hu Z, Butte AJ (2017). xCell: digitally portraying the tissue cellular heterogeneity landscape. Genome Biol..

[29] Hoshida Y (2008). Gene expression in fixed tissues and outcome in hepatocellular carcinoma. N. Engl. J. Med..

[30] Subramanian A (2005). Gene set enrichment analysis: a knowledge-based approach for interpreting genome-wide expression profiles. Proc. Natl Acad. Sci. USA.

[31] Pinyol R (2019). Molecular predictors of prevention of recurrence in HCC with sorafenib as adjuvant treatment and prognostic factors in the phase 3 STORM trial. Gut.

[32] Love MI, Huber W, Anders S (2014). Moderated estimation of fold change and dispersion for RNA-seq data with DESeq2. Genome Biol..

[33] Lin, D., Hiron, T. K. & O’Callaghan, C. A. Intragenic transcriptional interference regulates the human immune ligand MICA. *EMBO J.* **37**, e97138 (2018). 10.15252/embj.201797138 PMC5978299 29643123

[34] Cinghu S (2017). Intragenic enhancers attenuate host gene expression. Mol. Cell.

[35] Saxonov S, Berg P, Brutlag DL (2006). A genome-wide analysis of CpG dinucleotides in the human genome distinguishes two distinct classes of promoters. Proc. Natl Acad. Sci. USA.

[36] Illingworth RS (2010). Orphan CpG islands identify numerous conserved promoters in the mammalian genome. PLoS Genet..

[37] Singer GA (2008). Genome-wide analysis of alternative promoters of human genes using a custom promoter tiling array. BMC Genomics.

[38] Wang J, Ungar LH, Tseng H, Hannenhalli S (2007). MetaProm: a neural network based meta-predictor for alternative human promoter prediction. BMC Genomics.

[39] Ma X (2009). Systematic analysis of alternative promoters correlated with alternative splicing in human genes. Genomics.

[40] Roider HG, Lenhard B, Kanhere A, Haas SA, Vingron M (2009). CpG-depleted promoters harbor tissue-specific transcription factor binding signals—implications for motif overrepresentation analyses. Nucleic Acids Res..

[41] Cai SY, Yu D, Soroka CJ, Wang J, Boyer JL (2021). Hepatic NFAT signaling regulates the expression of inflammatory cytokines in cholestasis. J. Hepatol..

[42] Rach EA (2011). Transcription initiation patterns indicate divergent strategies for gene regulation at the chromatin level. PLoS Genet..

[43] Fenouil R (2012). CpG islands and GC content dictate nucleosome depletion in a transcription-independent manner at mammalian promoters. Genome Res.

[44] Bae S, Lesch BJ (2020). H3K4me1 distribution predicts transcription state and poising at promoters. Front. Cell Dev. Biol..

[45] Soares LM (2017). Determinants of histone H3K4 methylation patterns. Mol. Cell.

[46] Heintzman ND (2007). Distinct and predictive chromatin signatures of transcriptional promoters and enhancers in the human genome. Nat. Genet..

[47] Thomson JP (2010). CpG islands influence chromatin structure via the CpG-binding protein Cfp1. Nature.

[48] Kowalczyk MS (2012). Intragenic enhancers act as alternative promoters. Mol. Cell.

[49] Hughes AL, Kelley JR, Klose RJ (2020). Understanding the interplay between CpG island-associated gene promoters and H3K4 methylation. Biochim. Biophys. Acta Gene Regul. Mech..

[50] Taniguchi I, Iwaya C, Ohnaka K, Shibata H, Yamamoto K (2017). Genome-wide DNA methylation analysis reveals hypomethylation in the low-CpG promoter regions in lymphoblastoid cell lines. Hum. Genomics.

[51] Zilberman D, Coleman-Derr D, Ballinger T, Henikoff S (2008). Histone H2A.Z and DNA methylation are mutually antagonistic chromatin marks. Nature.

[52] Masalmeh RHA (2021). De novo DNA methyltransferase activity in colorectal cancer is directed towards H3K36me3 marked CpG islands. Nat. Commun..

[53] Jeziorska DM (2017). DNA methylation of intragenic CpG islands depends on their transcriptional activity during differentiation and disease. Proc. Natl Acad. Sci. USA.

[54] Baubec T (2015). Genomic profiling of DNA methyltransferases reveals a role for DNMT3B in genic methylation. Nature.

[55] Barski A (2007). High-resolution profiling of histone methylations in the human genome. Cell.

[56] Edmunds JW, Mahadevan LC, Clayton AL (2008). Dynamic histone H3 methylation during gene induction: HYPB/Setd2 mediates all H3K36 trimethylation. EMBO J..

[57] Smolle M (2012). Chromatin remodelers Isw1 and Chd1 maintain chromatin structure during transcription by preventing histone exchange. Nat. Struct. Mol. Biol..

[58] Sen P (2015). H3K36 methylation promotes longevity by enhancing transcriptional fidelity. Genes Dev..

[59] Zhou W (2018). DNA methylation loss in late-replicating domains is linked to mitotic cell division. Nat. Genet..

[60] Partridge EC (2020). Occupancy maps of 208 chromatin-associated proteins in one human cell type. Nature.

[61] Sarda S, Das A, Vinson C, Hannenhalli S (2017). Distal CpG islands can serve as alternative promoters to transcribe genes with silenced proximal promoters. Genome Res..

[62] Larsen F, Gundersen G, Lopez R, Prydz H (1992). CpG islands as gene markers in the human genome. Genomics.

[63] Karlsson K, Lonnerberg P, Linnarsson S (2017). Alternative TSSs are co-regulated in single cells in the mouse brain. Mol. Syst. Biol..

[64] Struhl K (2007). Transcriptional noise and the fidelity of initiation by RNA polymerase II. Nat. Struct. Mol. Biol..

[65] Ayoubi TA, Van De Ven WJ (1996). Regulation of gene expression by alternative promoters. FASEB J..

[66] Valcarcel LV (2021). Gene expression derived from alternative promoters improves prognostic stratification in multiple myeloma. Leukemia.

[67] Langmead B, Trapnell C, Pop M, Salzberg SL (2009). Ultrafast and memory-efficient alignment of short DNA sequences to the human genome. Genome Biol..

[68] Li H (2009). The Sequence Alignment/Map format and SAMtools. Bioinformatics.

[69] Amemiya HM, Kundaje A, Boyle AP (2019). The ENCODE blacklist: identification of problematic regions of the genome. Sci. Rep..

[70] Gao T, Qian J (2020). EnhancerAtlas 2.0: an updated resource with enhancer annotation in 586 tissue/cell types across nine species. Nucleic Acids Res..

[71] Ernst J (2011). Mapping and analysis of chromatin state dynamics in nine human cell types. Nature.

[72] Huang H (2019). Histone H3 trimethylation at lysine 36 guides m(6)A RNA modification co-transcriptionally. Nature.

[73] Goldman MJ (2020). Visualizing and interpreting cancer genomics data via the Xena platform. Nat. Biotechnol..

[74] Ramirez F (2016). deepTools2: a next generation web server for deep-sequencing data analysis. Nucleic Acids Res..

[75] Consortium EP (2012). An integrated encyclopedia of DNA elements in the human genome. Nature.

[76] Quinlan AR, Hall IM (2010). BEDTools: a flexible suite of utilities for comparing genomic features. Bioinformatics.

[77] Heinz S (2010). Simple combinations of lineage-determining transcription factors prime cis-regulatory elements required for macrophage and B cell identities. Mol. Cell.

[78] Liu J (2018). An integrated TCGA Pan-cancer clinical data resource to drive high-quality survival outcome analytics. Cell.

