# Supplementary material for: Alternative promoters in CpG depleted regions are prevalently associated with epigenetic misregulation of liver cancer transcriptomes
Source: Nat Commun. 2023 May 11;14:2712. doi: 10.1038/s41467-023-38272-4 (PMC10175279; doi:10.1038/s41467-023-38272-4)
Supplement: Supplementary file 1 — Supplementary Information [file 41467_2023_38272_MOESM1_ESM.pdf]

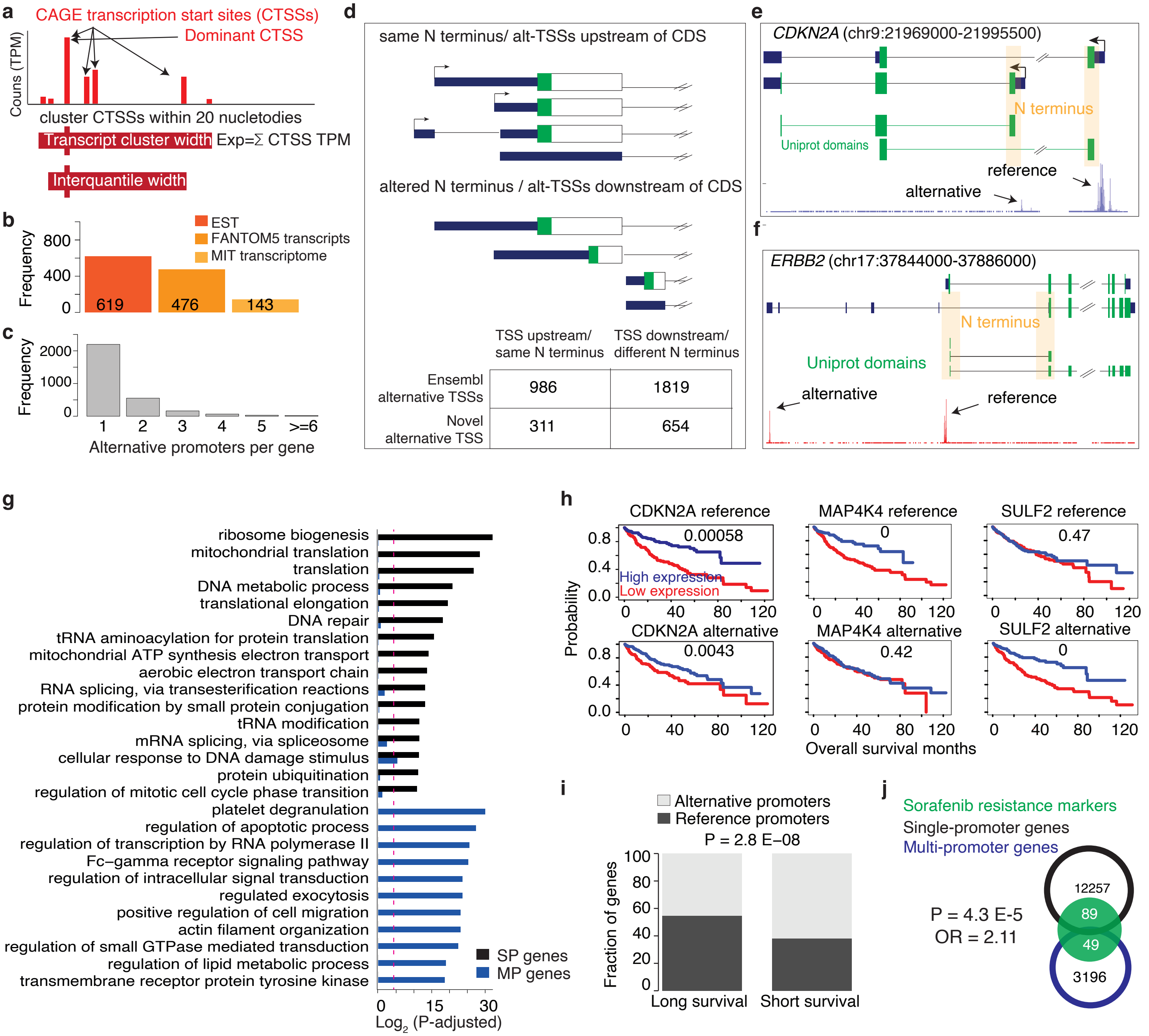

Supplementary Figure 1. Annotation of alternative promoters in hepatocellular carcinoma patients. (**a**) A schematic workflow of clustering proximal CAGE transcription start sites (CTSSs) into transcript clusters (TCs). The height of CTSSs determines the frequency of mapped reads that are quantified in tags per million (TPM). The expression of TCs is determined by the sum of CTSSs. The interquartile widths of TCs are determined by 0.1 to 0.9 fraction of expression levels. (**b**) Barplots show the number of novel alternative TSSs that are supported by transcripts. (**c**) Barplots showing the number of alternative promoters for each multi-promoter genes. (**d**) Schematic representation and distribution of alternative promoter TSSs in regards to coding sequence start. (**e-f**) A UCSC browser view of *CDKN2A* and *ERBB2* genes with UniProt domains and CAGE tags. Alternative promoters miss N terminus of UniProt domains. (**g**) Enriched gene ontology terms associated with single-promoter (SP) and multi-promoter (MP) genes. P-values were computed using two-sided Fisher's exact test and corrected for multiple testing. (**h**) Individual examples of Kaplan-Meier overall survival analysis for expression of reference and alternative promoters. (**i**) Classification of survival-associated genes based on time to death (long/short survival time) for reference and alternative promoters. Two-sided Fisher's exact test was used to compute P value. (**j**) Overlap of sorafenib resistance marker-genes with single and multi-promoter genes. Two-sided Fisher's exact test was used to compute P value.

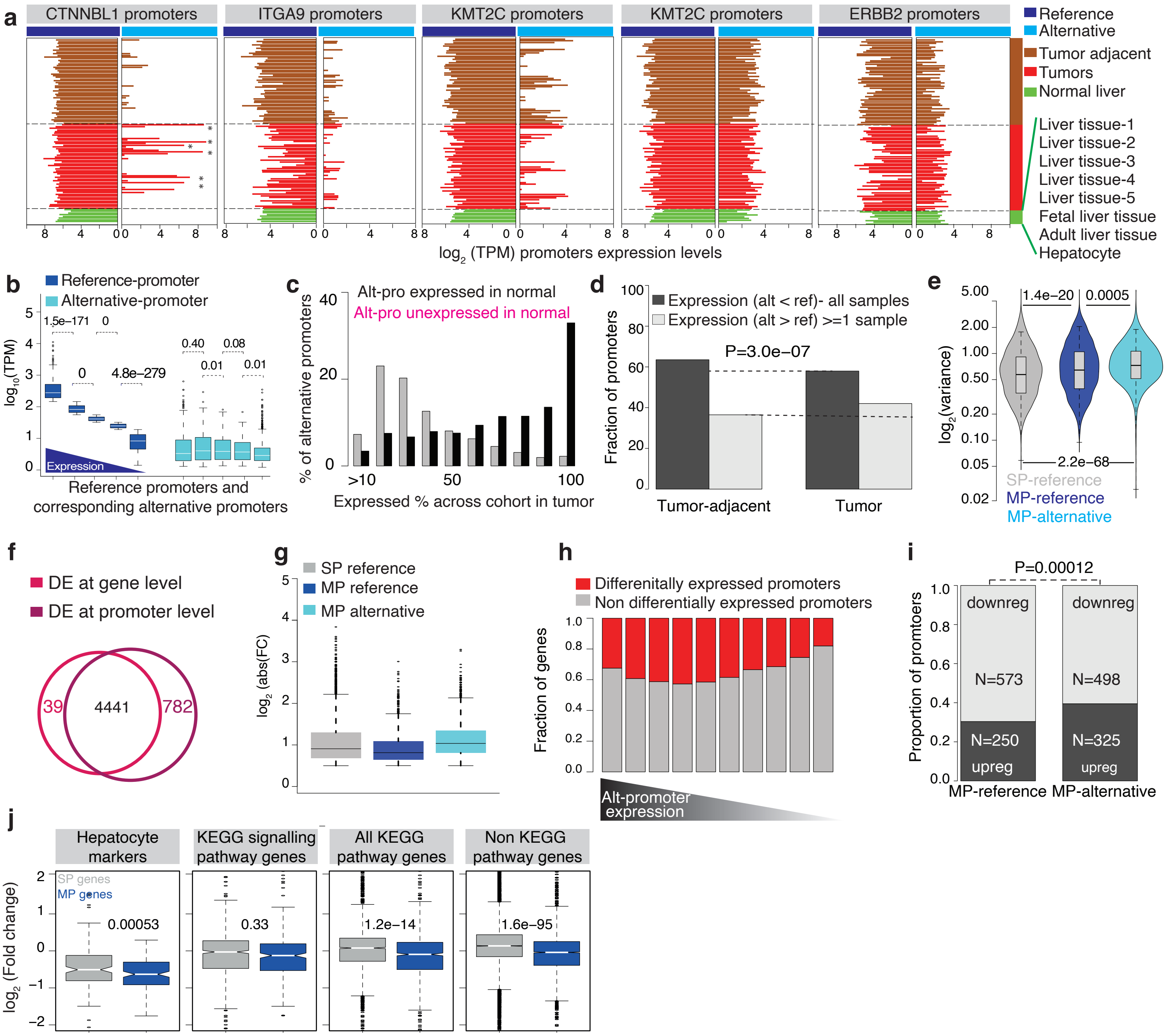

Supplementary Figure 2. Expression dynamics of reference and alternative promoters. (**a**) Expression of reference and alternative promoters (n=50 tumors, n=tumor-adjacent tissues and n=8 normal liver tissues). The asterisk indicates alternative promoter has higher expression in a given sample. The gene KMT2C has two alternative promoters. (**b**) Expression levels of reference promoters divided into 5 bins (N= 585 promoters in each bin) and their corresponding expression levels of alternative promoters in 5 bins (N=965, N=848, N=793, N=760 and N=713 promoters). P values were computed using the two-sided t test. (**c**) Distribution of alternative promoters expressed across the cohort. Alternative promoters were grouped based on the presence or absence of its expression in the normal liver tissues. X-axis indicates the percentage a promoter is expressed across the cohort. (**d**) Distribution of expression levels of reference and alternative promoters at individual samples. P-value was computed using two sided Fisher's exact test. (**e**) Violin plots show the distribution of variance of expression levels of single promoter (SP) reference (n=12493), multi promoter (MP) reference (n=2926) and alternative (n=4083) promoters. P values were computed using the two-sided t test. Boxplots show the 5th, 25th, 50th, 75th, and 95th percentiles where center line is the median. (**f**) Venn diagram represents the overlap of differentially expressed genes and promoters between tumor and tumor-adjacent tissues. (**g**) Distribution of absolute fold-change levels of differentially expressed SP reference (n=3625), MP reference (n=896) and MP alternative (n=1376) promoters. (**h**) Distribution of differentially expressed alternative promoters ranked in different bins based on decreasing expression levels. Each bin contains 400 promoters. (**i**) Distribution of differentially upregulated and downregulated reference/alternative promoters from multi promoter genes. P-value was computed using two-sided Fisher's exact test. (**j**) Expression fold change between tumor and tumor adjacent tissues for SP and MP genes across different functional classification of genes. P values were computed using the two sided t test. All boxplots show the 5th, 25th, 50th, 75th, and 95th percentiles where center line is the median.

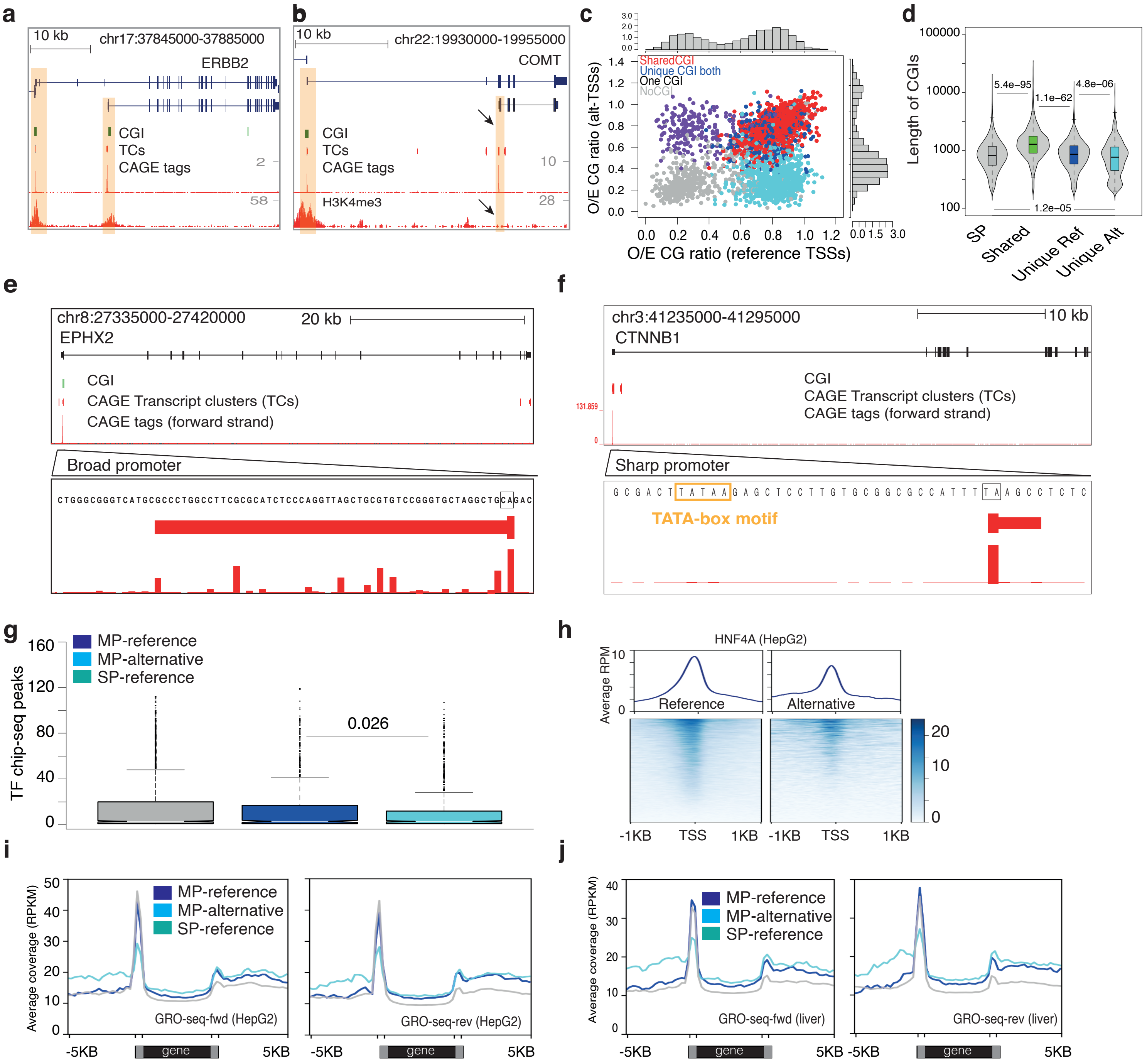

Supplementary Figure 3. Sequence and motif enrichment of reference and alternative promoters. (**a-b**) A UCSC browser screenshot of ERBB2 and COMT gene along with tracks of CpG islands (CGI), CAGE-seq and H3K4me3. (**c**) Scatter plot showing the observed/expected (O/E) ratio of CG dinucleotides around reference and alternative promoters. (**d**) Length of CGIs overlapping single promoters (SP, n=8949), CGIs shared by reference and alternative promoters (shared; n=1018), unique reference promoters (n=1252) and unique alternative promoters (n=705). P values were computed using two-sample t-tests. Boxplots show the 5th, 25th, 50th, 75th, and 95th percentiles where center line is the median. (**e-f**) A UCSC browser shot of *CTNNB1* and *EPHX2* genes with CAGE tags, transcript clusters and CGI tracks. The zoomed view shows transcript clusters width and sequences around dominant transcription start site (TSS). The *CTTNB1* gene has sharp promoter with one dominant TSS and TATA box motif in upstream region. The *EPHX2* gene has broad promoter with multiple TSSs and lack TATA box motif. (**g**) Frequency of ENCODE HepG2 transcription factors peaks overlapping on multi-promoter (MP) reference (n=761), MP alternative promoters (n=974) and single promoter (SP) reference promoter (n=3026). Boxplots show the 5th, 25th, 50th, 75th, and 95th percentiles where center line is the median. P values were computed using the two-sample t-test. (**h**) Enrichment of HNF4A transcription factor centered at transcription start sites. (**i-j**) The coverage of nascent RNA reads from GRO-seq in HepG2 (i) cells and liver cells (j) for genes in forward and reverse strand.

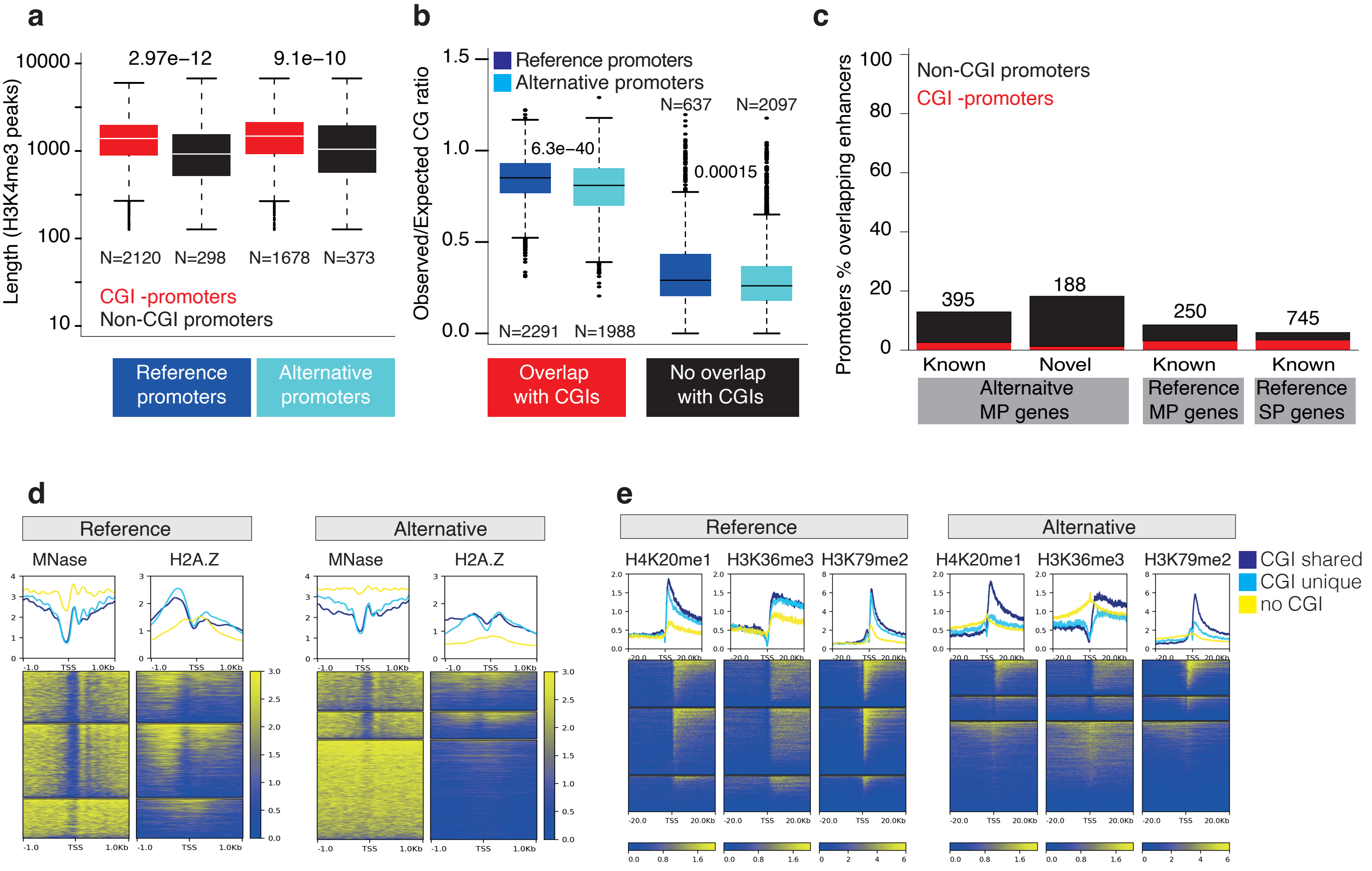

Supplementary Figure 4. Histone modifications around CpG island (CGI) enhancers, nonCGI enhancers along with reference and alternative promoters. (**a**) Distribution of length of H3K4me3 peaks from HCC patient across reference and alternative promoters. Promoters are grouped based on overlap with CGIs. Boxplots show the 5th, 25th, 50th, 75th, and 95th percentiles where center line is the median. P value was computed using two-sided tests. (**b**) Observed/Expected ratio of CG dinucleotide across reference and alternative promoters grouped based on overlap with CGIs. Two-sided t test was used to calculate p-values. Boxplots show the 5th, 25th, 50th, 75th, and 95th percentiles where center line is the median. (**c**) Distribution of overlap HepG2 annotated chromHMM enhancers with Ensembl annotated (reference and alternative) promoters and novel alternative promoters. (**d**) Line plots and heatmaps show MNase and H2A.Z levels across reference and alternative promoters on HepG2 cells.. Heatmaps on the botton show expression levels for each enahncers. (**e**) Average coverage of histones (H4K20me1, H3K26me3 and H3K79me2) along reference and alternative promoters.

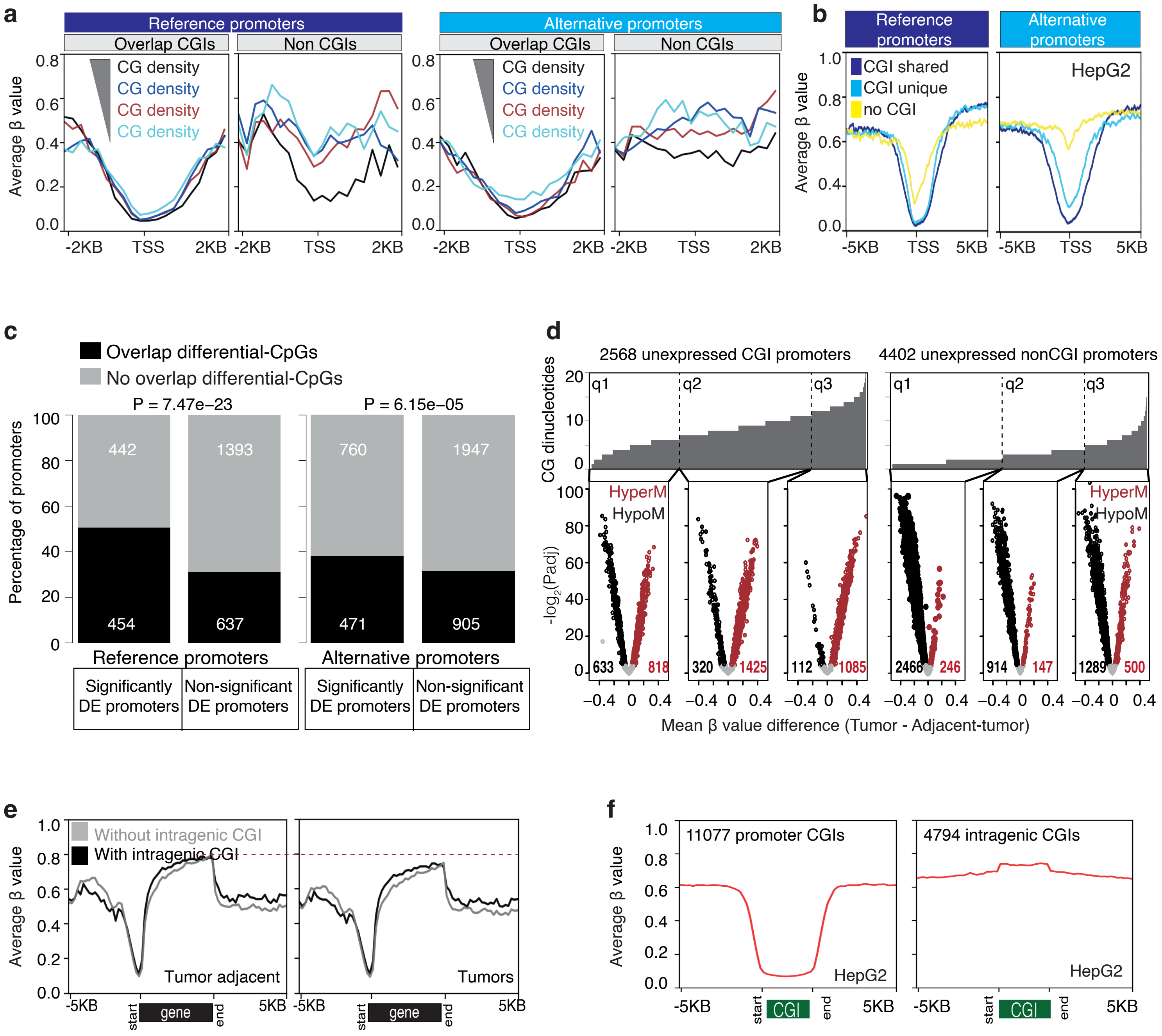

Supplementary Figure 5. DNA methylation landscape around transcription start sites (TSSs) and gene body. (**a**) Average methylation level (β values) across TSSs of CGI and nonCGI promoters across TCGA HCC (n=379) tumors. Promoters are grouped into four bins based on CG density around TSSs. (**b**) Average methylation level from reduced representation bisulfite sequencing (RRBS) on HepG2 cells across reference and alternative promoters that are grouped based on overlap with CpG islands (CGIs). (**c**) Overlap of differentially expressed (DE) reference and alternative promoters with differentially methylated (DM) CpGs in a 500 nucleotides window around TSSs. P values were computed using two sided Fisher's exact test. (**d**) Differentially methylated CpGs in promoter regions of genes unexpressed in HCC. Genes overlapping with CGIs are separated from nonCGI genes. Barplots (top panel) show CG dinucleotides frequency in a 100-nucleotide window which are grouped into three quartiles (q1, q2, q3) based on CG dinucleotides frequency. The scatter plots (bottom panel) show differentially hypermethylated (brown) and hypomethylated (black) CpGs. P-values were determined by two-tailed unpaired t-tests between HCC tumors (n=379) and tumor adjacent tissues (n=51). P values were adjusted for multiple testing. (**e**) Average methylation level across genes body in tumor adjacent tissues (n=51; left panel) and HCC tumor (n=379; right panel). Genes are classified into two groups based on presence or absence of intragenic CGIs. (**f**) Mean methylation levels along promoter CGIs (left panle) and intragenic CGIs (right panel) on HepG2 cells. CGIs of varying length are scaled between start and end.

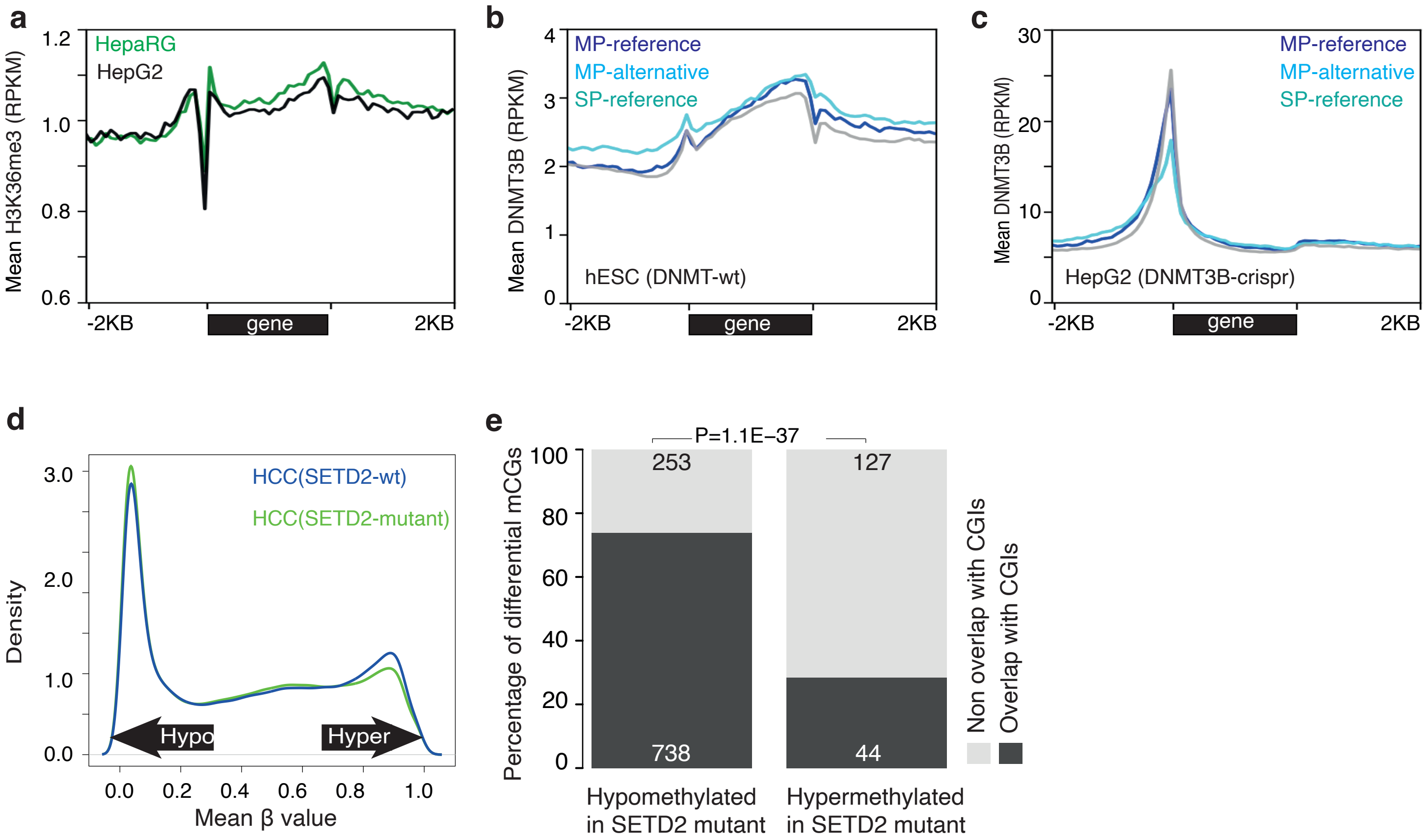

Supplementary Figure 6. Genomic coverage of H3K36me3 and DNMT3B across intragenic regions. (**a**) The average coverage of H3K36me3 along the gene body and flanking regions in HepG2 cells and HepaRG cells (normal hepatocytes). (**b**) The average coverage of DNMT3B across the gene body in human embryonic stem cells (hESC). (**c**) The average coverage of DNMT3B across the gene body in CRISPR epitope tagging (through insertion) of DNMT3B in HepG2 cells. (**d**) The density of mean methylation levels (β values) of TCGA HCC patients with SETD2 mutants (n=15) and SETD2 wildtype (n=362). (**e**) Boxplots show the distribution of hypermethyated and hypomethylated CpGs (in SETD2-mutant versus SETD2 wild-type tumors) overlapping CpG islands (CGIs). Two-sided Fisher's exact test was used to calculate p-value.
