# Supplementary material for: Alternative promoters in CpG depleted regions are prevalently associated with epigenetic misregulation of liver cancer transcriptomes
Source: Nat Commun. 2023 May 11;14:2712. doi: 10.1038/s41467-023-38272-4 (PMC10175279; doi:10.1038/s41467-023-38272-4)
Supplement: Supplementary file 2 — Reporting Summary [file 41467_2023_38272_MOESM2_ESM.pdf]

nature portfolio

Corresponding author(s): Chirag Nepal and Jesper B Andersen

Last updated by author(s): 04/04/2023

Reporting Summary

Nature Portfolio wishes to improve the reproducibility of the work that we publish. This form provides structure for consistency and transparency in reporting. For further information on Nature Portfolio policies, see our Editorial Policies and the Editorial Policy Checklist.

Statistics

For all statistical analyses, confirm that the following items are present in the figure legend, table legend, main text, or Methods section.

n/a	Confirmed	
☐	☒	The exact sample size (*n*) for each experimental group/condition, given as a discrete number and unit of measurement
☐	☒	A statement on whether measurements were taken from distinct samples or whether the same sample was measured repeatedly
☐	☒	The statistical test(s) used AND whether they are one- or two-sided<br>*Only common tests should be described solely by name; describe more complex techniques in the Methods section.*
☒	☐	A description of all covariates tested
☐	☒	A description of any assumptions or corrections, such as tests of normality and adjustment for multiple comparisons
☒	☐	A full description of the statistical parameters including central tendency (e.g. means) or other basic estimates (e.g. regression coefficient) AND variation (e.g. standard deviation) or associated estimates of uncertainty (e.g. confidence intervals)
☐	☒	For null hypothesis testing, the test statistic (e.g. *F*, *t*, *r*) with confidence intervals, effect sizes, degrees of freedom and *P* value noted<br>*Give P values as exact values whenever suitable.*
☒	☐	For Bayesian analysis, information on the choice of priors and Markov chain Monte Carlo settings
☐	☒	For hierarchical and complex designs, identification of the appropriate level for tests and full reporting of outcomes
☐	☒	Estimates of effect sizes (e.g. Cohen's *d*, Pearson's *r*), indicating how they were calculated

Our web collection on statistics for biologists contains articles on many of the points above.

Software and code

Policy information about availability of computer code

Data collection	Analyzed data are collected from public repositories, HCC CAGE (dbGap: phs000885.v1.p1), normal liver tissues CAGE (DDBJ DRA: DRA000991), HCC histone modifications and RRBS (GEO: GSE112221), H1ESC DNMT3B (GEO: GSE150072), HepG2 shCtrl H3K36me3 and shSETD2 H3K36me3 (GEO: GSE110323). The data generated by the TCGA were downloaded from https://xenabrowser.net/ and ENCODE data downloaded from https://www.encodeproject.org
Data analysis	Data were analyzed by using publicly available softwares,which are cited in the paper. The version of software and syntax used are listed in the paper.

For manuscripts utilizing custom algorithms or software that are central to the research but not yet described in published literature, software must be made available to editors and reviewers. We strongly encourage code deposition in a community repository (e.g. GitHub). See the Nature Portfolio guidelines for submitting code & software for further information.

Data

Policy information about availability of data

All manuscripts must include a data availability statement. This statement should provide the following information, where applicable:

- Accession codes, unique identifiers, or web links for publicly available datasets
- A description of any restrictions on data availability
- For clinical datasets or third party data, please ensure that the statement adheres to our policy

Sequencing data analyzed in this study are publicly available from previous studies. Data accession codes are provided. The data used in this study are available under accession number (HCC CAGE data from dbGap: phs000885.v1.p1), normal liver tissues CAGE (DDBJ DRA: DRA000991), histone modifications and RRBS of HCC patients' (GEO: GSE112221), H1ESC DNMT3B (GEO: GSE150072), HepG2 shCtrl H3K36me3 and shSETD2 H3K36me3 (GEO: GSE110323). The data generated by the TCGA (https://www.cancer.gov/tcga) were downloaded from https://xenabrowser.net/ and ENCODE data downloaded from https://www.encodeproject.org. Processed data are provided as supplementary tables. Source data are provided with this paper.

Human research participants

Policy information about studies involving human research participants and Sex and Gender in Research.

Reporting on sex and gender	N/A
Population characteristics	N/A
Recruitment	N/A. All public data were used.
Ethics oversight	N/A

Note that full information on the approval of the study protocol must also be provided in the manuscript.

Field-specific reporting

Please select the one below that is the best fit for your research. If you are not sure, read the appropriate sections before making your selection.

☒ Life sciences ☐ Behavioural & social sciences ☐ Ecological, evolutionary & environmental sciences

For a reference copy of the document with all sections, see nature.com/documents/nr-reporting-summary-flat.pdf

Life sciences study design

All studies must disclose on these points even when the disclosure is negative.

Sample size	No statistical methods were used to predetermine sample size. We used multiple large HCC cohorts. Collectively, data analyzed in this work represent one of the largest sample size with different sequencing data.
Data exclusions	No data were excluded from the analyses.
Replication	Our findings are reproduced across different HCC cohorts. We reproduced findings from HCC patients in HepG2 cell lines.
Randomization	No randomization was done as we focus to understand differences between tumor and matched non tumor tissues .
Blinding	N/A. No clinical trials done, as we compare differences between tumor and matched non tumor tissues.

Reporting for specific materials, systems and methods

We require information from authors about some types of materials, experimental systems and methods used in many studies. Here, indicate whether each material, system or method listed is relevant to your study. If you are not sure if a list item applies to your research, read the appropriate section before selecting a response.

Materials & experimental systems

n/a	Involved in the study
☒	☐ Antibodies
☐	☒ Eukaryotic cell lines
☒	☐ Palaeontology and archaeology
☒	☐ Animals and other organisms
☒	☐ Clinical data
☒	☐ Dual use research of concern

Methods

n/a	Involved in the study
☐	☒ ChIP-seq
☒	☐ Flow cytometry
☒	☐ MRI-based neuroimaging

Clinical data

Policy information about clinical studies

All manuscripts should comply with the ICMJE guidelines for publication of clinical research and a completed CONSORT checklist must be included with all submissions.

Clinical trial registration	N/A
Study protocol	N/A

Data collection	Use publicly available clinical data from TCGA, downloaded from UCSC Xena browser.
Outcomes	N/A

ChIP-seq

Data deposition

☒ Confirm that both raw and final processed data have been deposited in a public database such as GEO.

☒ Confirm that you have deposited or provided access to graph files (e.g. BED files) for the called peaks.

Data access links<br>*May remain private before publication.*	Data analyzed in the study have their GEO accession ID provided in the manuscript.
Files in database submission	N/A
Genome browser session<br>(e.g. UCSC)	N/A

Methodology

Replicates	Pre defined Chip-seq peaks from ENCODE were downloaded and analyzed in the study.
Sequencing depth	N/A
Antibodies	N/A
Peak calling parameters	N/A
Data quality	N/A
Software	N/A
